# Dual Chloride Confinement in Noble Metal‐Doped NiV LDH Catalysts Enables Stable Industrial-Level Seawater Electrolysis

**DOI:** 10.1007/s40820-026-02067-1

**Published:** 2026-01-16

**Authors:** Kai Liu, Yaohai Cai, Xiaotian Wei, Lihang Qu, Jianxi Lu, Yingwei Qi, Zhenbo Wang, Dong Liu

**Affiliations:** 1 Guangdong Provincial Key Laboratory of New Energy Materials Service Safety, College of Materials Science and Engineering, Shenzhen University, Shenzhen, 518060 People’s Republic of China; 2 Key Laboratory of Space Power-Sources, MIIT Key Laboratory of Critical Materials Technology for New Energy Conversion and Storage, MOE Engineering Research Center for Electrochemical Energy Storage and Carbon Neutrality in Cold Regions, School of Chemistry and Chemical Engineering, Harbin Institute of Technology, Harbin, 150001 People’s Republic of China

**Keywords:** Seawater electrolysis, Chloride confinement, NiV LDH, Noble metal doping, Long-term stability

## Abstract

Seawater electrolysis is an appealing route toward sustainable hydrogen production, yet its practical deployment is hindered by severe chloride-induced corrosion and parasitic chlorine oxidation. Here, we report noble metal-doped NiV layered double hydroxides (LDHs) that integrate electronic modulation with a dual chloride confinement mechanism. Ir incorporation simultaneously establishes strong Ir-Cl coordination and dynamically regenerated VO_4_^3−^ layers, producing an adaptive electrostatic shield that effectively suppresses chloride penetration. As a result, Ir-NiV LDH delivers nearly 100% oxygen evolution reaction selectivity and outstanding stability over 2750 h at 500 mA cm^−2^. Meanwhile, Ru doping optimizes the hydrogen evolution pathway, enabling a low overpotential of 195 mV and >2350 h durability. When paired in a twso-electrode electrolyzer, the Ru-NiVLDH||Ir-NiVLDH system exhibits industrial-level performance and unprecedented robustness in alkaline seawater. This dual chloride confinement concept provides a general framework for catalyst design in corrosive ionic environments, extending beyond seawater splitting toward other electrochemical energy conversion processes.

**Supplementary Information:**

The online version contains supplementary material available at 10.1007/s40820-026-02067-1.

## Highlights

- Noble metal doping into NiV-layered double hydroxides optimizes the electronic structure of active sites, significantly enhancing its catalytic performance for the hydrogen evolution reaction and oxygen evolution reaction.
- A “dual chloride confinement” strategy is proposed to overcome chloride corrosion in seawater electrolysis by synergizing strong adsorption (Ir-Cl) with electrostatic repulsion (VO_4_^3−^).
- Offers a practical route toward economically viable and sustainable hydrogen production from seawater.

**Supplementary Information:**

The online version contains supplementary material available at 10.1007/s40820-026-02067-1.

## Graphical abstract

**Figure:**
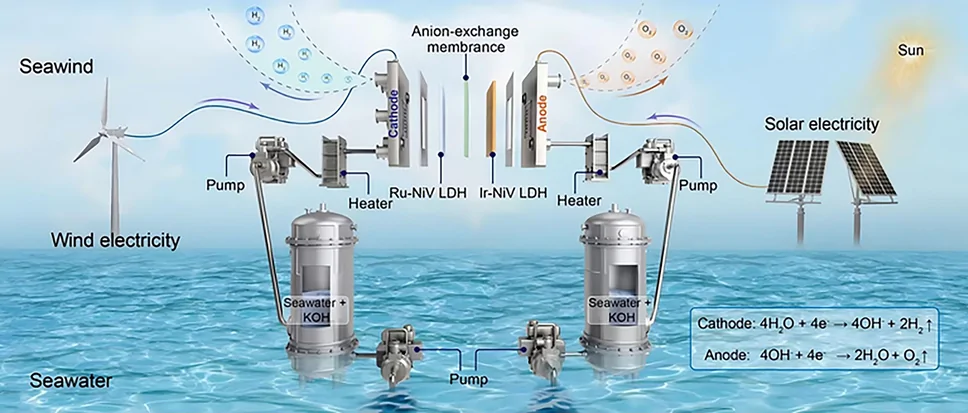

## Introduction

Electrochemical water splitting powered by renewable energy is regarded as a cornerstone for sustainable hydrogen production at scale [1]. However, current electrolyser technologies rely predominantly on high-purity freshwater, which constrains deployment in water-scarce regions. The prospect of coupling electrolysis with offshore or coastal renewable resources is particularly attractive, yet the scarcity of freshwater underscores the need to directly utilize seawater as the feedstock [2–4] Seawater constitutes ~96.5% of the Earth’s water reserves and is virtually inexhaustible, but its chemical complexity imposes formidable challenges [5]. The high concentration of chloride ions (~0.5 M) reduces the thermodynamic potential window by ~490 mV under alkaline conditions, favoring parasitic chlorine oxidation reaction (ClOR) over oxygen evolution reaction (OER) [6], while simultaneously accelerating catalyst degradation through chloride adsorption and corrosion [7]. Overcoming these issues to achieve catalysts that are simultaneously active, durable, and highly selective in seawater remains one of the central challenges in advancing electrochemical hydrogen production [8].

To address this problem, extensive research has focused on catalyst design strategies such as heterojunction engineering [9–11], heteroatom doping [12, 13], and defect engineering [14–18]. Among these, noble metal doping has emerged as a particularly versatile approach, capable of tuning electronic structures and tailoring intermediate adsorption energies [19, 20]. For example, Ru-doped layered double hydroxides (LDHs) have been shown to accelerate hydrogen evolution by strain-induced modulation of the d-band center, thereby enhancing reaction kinetics [21]. Such examples highlight the potential of noble metal doping as a general platform for optimizing seawater electrocatalysis. Equally critical, however, is the suppression of ClOR by restricting chloride access to active sites [22, 23]. Existing approaches typically rely on either physical exclusion, where protective barriers reduce chloride transport, or chemical confinement, where anion-rich layers or specific adsorption motifs repel chloride ions [24, 25]. While chemical confinement generally provides better selectivity and durability [26], most reported strategies rely on a single confinement mechanism [27], which often collapses under the high current densities necessary for practical electrolysis due to concentration gradients and chloride penetration [28].

Here, we introduce a dual chloride confinement strategy realized in noble metal doped NiV LDHs that combines electronic modulation with adaptive chloride management. Ir incorporation establishes robust Ir-Cl coordination alongside dynamically regenerated VO_4_^3−^ species, together forming a dual repelling interface that ensures nearly 100% OER selectivity and outstanding stability exceeding 2750 h at industrial current densities. In parallel, Ru incorporation optimizes the hydrogen evolution pathway, achieving low overpotentials (195 mV at 500 mA cm^−2^) and durability beyond 2350 h. When integrated into a commercial electrolyzer, the system not only exhibits excellent performance, but also enables long-term operation in alkaline seawater. More broadly, this work establishes a conceptual framework for chloride management and outlines a feasible pathway for the scalable, selective, and cost-competitive conversion of seawater to hydrogen.

## Experimental Section

### Synthesis of NiV LDH

The NiV LDH catalysts was fabricated by a hydro-thermal synthesis method. NF (2 × 3 cm^2^) was washed with diluted hydrochloric acid, acetone, deionized water, and ethanol for 10 min. To prepare the solution, 0.6 mmol of Ni(NO_3_)_2_·6H_2_O, 0.48 mmol of VCl_3_ and 4 mmol urea were combined in 30 mL of deionized water and stirred for 30 min. This mixture was then transferred to a 50 mL Teflon-lined stainless-steel autoclave. The autoclave drying box was heated to 120 °C for 6 h. Subsequently, the samples were removed, rinsed with deionized water, and dried to yield NiV LDH.

### Synthesis of Ru-NiV LDH Ir-NiV LDH Os-NiV LDH and Pt-NiV LDH

The Ru-NiV LDH, Ir-NiV LDH, Os-NiV LDH, and Pt-NiV LDH were obtained by the same approach as the preparation of NiV LDH, expect that the solution was added with 0.24 mmol of IrCl_3_·xH_2_O, RuCl_3_·xH_2_O, OsCl_3_, and H_2_PtCl_6_, respectively.

### Synthesis of RuO_2_ and Pt/C Catalysts on Ni Foam

The 20 mg of Pt/C(RuO_2_), 60 μL Nafion, 540 μL deionized water, and 400 μL anhydrous ethanol was mixed. The above mixed slurry was ultrasonically treated for 30 min. The 200 μL solution dropwise to 1.5 × 1.0 cm^2^ NF, and then the NF was placed under a baking lamp for 15 min.

### Physical Characterizations

The morphologies and dimensions of the catalysts were measured using scanning electron microscopy (SEM) analyses (Hitachi S-8200). Transmission electron microscopy (TEM) and high-resolution transmission electron microscopy (HR-TEM) images were captured with a JEM2100UHR instrument. Crystal structures were determined by powder X-ray diffraction (X’Pert PRO MPD system) operating at 40 kV and 30 mA with Cu Kα radiation (λ = 1.5418 Å). X-ray photoelectron spectroscopy (XPS) assessments were carried out using an AXIS SUPRA equipped with a monochromatic Al Kα source at 15 mA and 14 kV. XPS spectra were calibrated against the carbon 1s peak, setting the main line to 284.6 eV. In situ Raman spectra were collected with an Invia Qontor Raman spectroscopy system utilizing a 532 nm laser (The chemicals, electro chemical measurements and calculation details, and density functional theory (DFT) methodology can be seen in the Supporting Information).

## Results

### Synthesis and Characterizations

**Fig. 1:**
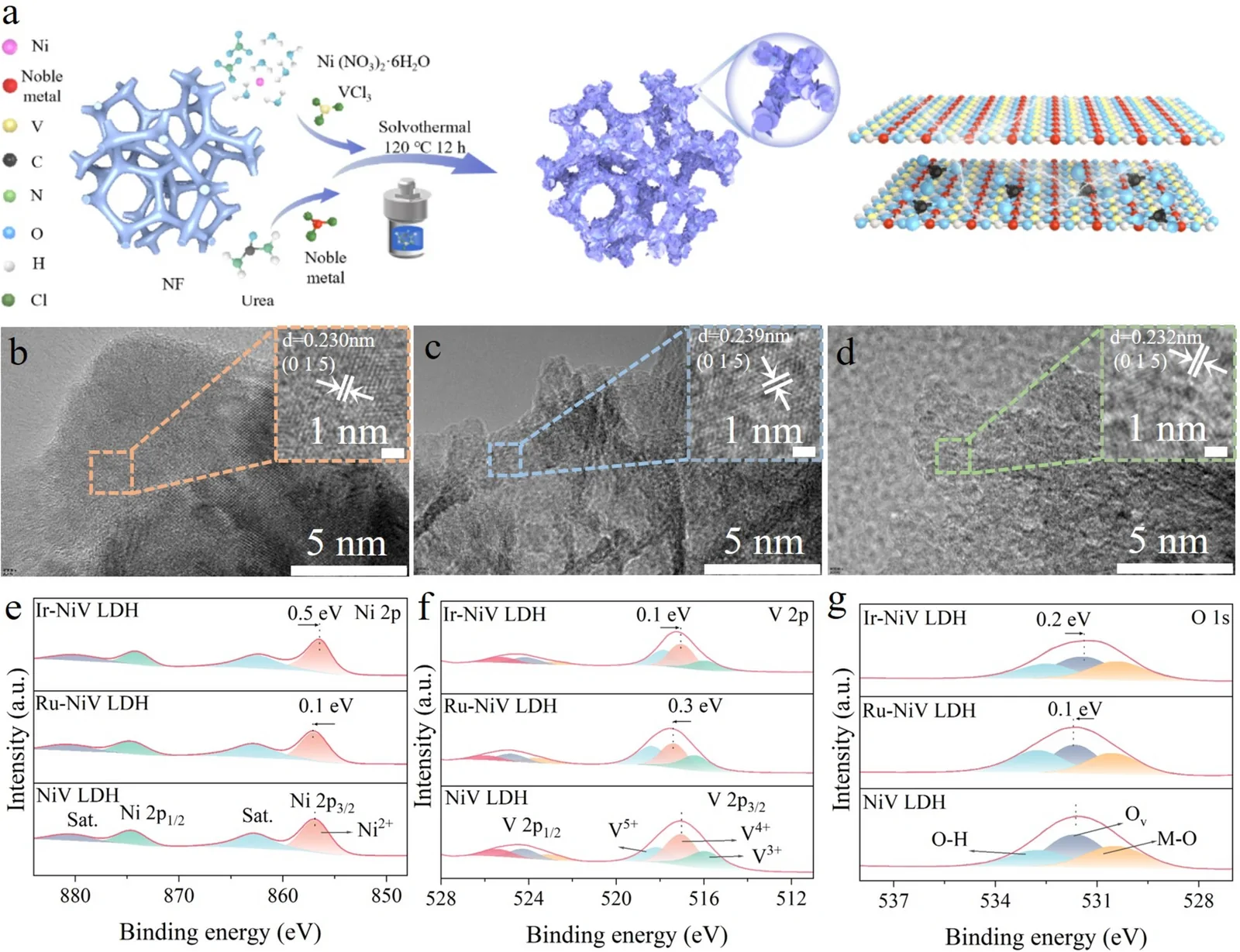
**a** Schematic illustration of the preparation of noble metal-doped NiV LDH, TEM images of **b** NiV LDH, **c** Ru-NiV LDH, **d** Ir-NiV LDH, XPS spectra of NiV LDH, Ru-NiV LDH, and Ir-NiV LDH of **e** Ni 2*p*, **f** V 2*p*, **g** O 1*s*

The preparation of noble metal (e.g., Ru, Ir, Pt, Os) doped NiV LDH catalysts on nickel foam (NF) using a one-step hydrothermal method aims to simultaneously modulate the electronic structure of NiV LDH (Fig. 1a), thereby enhancing the activity, stability, and selectivity of the catalyst. Among a series of noble metal dopants, Ru-doped NiV LDH demonstrates exceptional HER performance, while Ir-doped NiV LDH exhibits superior OER performance. Consequently, the subsequent step involves characterizing these two outstanding catalysts along with the NiV LDH. The nanostructure of the noble metal-doped NiV LDH was investigated using scanning electron microscopy (SEM) and transmission electron microscopy (TEM). SEM images of as-prepared sample exhibit a layered morphology with numerous layered nanosheet structure (Fig. S1). And, TEM images of NiV LDH, Ru-NiV LDH, and Ir-NiV LDH clearly revealed the layered nanosheet structure of the catalysts (Fig. S2). High-resolution TEM (HR-TEM) images of NiV LDH, Ru-NiV LDH, and Ir-NiV LDH displayed lattice fringe spacings of 0.230, 0.239, and 0.232 nm (Fig. 1b-d), respectively, corresponding to the (015) plane (Fig. S3). Compared to undoped NiV LDH, Ru-NiV LDH, and Ir-NiV LDH achieved lattice strain of 3.91 and 0.87%, respectively, suggesting successful incorporation of noble metals [29, 30]. Selected area electron diffraction (SAED) patterns of NiV LDH, Ru-NiV LDH, and Ir-NiV LDH clearly identified the (110), (015), and (009) crystal planes of NiV LDH (Fig. S2b, d, and f). Energy-dispersive X-ray spectroscopy (EDX) elemental mapping of the HR-TEM images confirmed the uniform distribution of elements in NiV LDH, Ru-NiV LDH, and Ir-NiV LDH, further verifying the successful incorporation of Ru and Ir into the NiV LDH structure (Figs. S5-S7). TEM-EDX analysis revealed that the noble metal content in Ru-NiV LDH and Ir-NiV LDH catalysts was 5.58 wt% Ru and 7.62 wt% Ir (Fig. S4), respectively. In contrast, inductively coupled plasma optical emission spectrometry (ICP-OES) analysis reported lower Ru and Ir contents of 0.23 and 0.68 wt% (Table S1), respectively. This discrepancy arises because ICP-OES analyzes the entire sample, including the nickel foam substrate, resulting in a relatively lower noble metal content compared to the localized TEM-EDX analysis. These results collectively confirm the successful doping of Ru and Ir into the NiV LDH.

X-ray diffraction (XRD) pattern analysis confirmed the crystal structure of the prepared samples (Fig. S8a), with diffraction peaks matching standard cards for Ni (JCPDS 000-004-0850) and NiV LDH (JCPDS 000-052-1627), in agreement with HR-TEM results [31, 32]. Notably, the diffraction peaks corresponding to the (110) plane shifted to lower angles (Fig. S8b), confirming the successful doping of noble metals into the NiV LDH lattice, which is consistent with the HR-TEM characterization. X-ray photoelectron spectroscopy (XPS) further revealed the surface composition and chemical states of NiV LDH, Ru-NiV LDH, and Ir-NiV LDH (Figs. 1e-g and S9). Full-spectrum XPS analysis indicated the presence of Ni, V, and O in NiV LDH, Ru, Ni, V, and O in Ru-NiV LDH, and Ir, Ni, V, and O in Ir-NiV LDH. In the high-resolution Ni 2*p* XPS spectrum (Fig. 1e), at the peaks at 856.9 and 874.5 eV are characteristic peaks of Ni^2+^, accompanied by satellite peaks at 861.9 and 880.0 eV [33]. Compared with the undoped NiV LDH, the Ni 2*p* binding energies of Ru-NiV LDH and Ir-NiV LDH shift by approximately 1.1 and 0.6 eV to lower energy, indicating successful noble metal doping and modulation of the NiV LDH electronic structure. The large electron offset may be caused by the change of surface electron density after noble metal doping [34]. In the V 2*p* spectrum (Fig. 1f), three characteristic peaks at 516.2, 517.4, and 519.0 eV, corresponding to V^3+^, V^4+^, and V^5+^ species [35], respectively, energies shifted by 1.2 and 0.2 eV in Ru-NiV LDH and Ir-NiV LDH, further evidencing strong electronic interactions at the interface. The high-resolution O 1*s* spectrum of NiV LDH deconvolutes into at 530.7 eV (M-O bonds), 531.9 eV (oxygen vacancies), and 533.5 eV (adsorbed oxygen) (Fig. 1g) [21]. In Ru-NiV LDH and Ir-NiV LDH, these peaks shift to lower energies by 1.1 and 0.7 eV, respectively, reinforcing the evidence of interfacial electronic interactions. Electron paramagnetic resonance (EPR) indicates that while the oxygen vacancy content remains similar to NiV LDH, a high density of vacancies provides additional active sites with unsaturated electronic structures (Fig. S11). The high-resolution Ru 3*p* spectrum of Ru-NiV LDH (Fig. S10a) shows peaks at 463.6 and 485.8 eV, confirming the presence of Ru^3+^ [36]. Similarly, the high-resolution Ir 4*f* spectra of Ir-NiV LDH at 62.7 and 65.8 eV, corresponding to Ir^3+^ (Fig. S10b), confirmed the successful incorporation of Ir [33, 37]. These results, further supported by ICP-OES, TEM, and TEM-EDX, demonstrate a uniform distribution of Ru and Ir in the doped catalysts.

### Electrocatalytic Performance

**Fig. 2:**
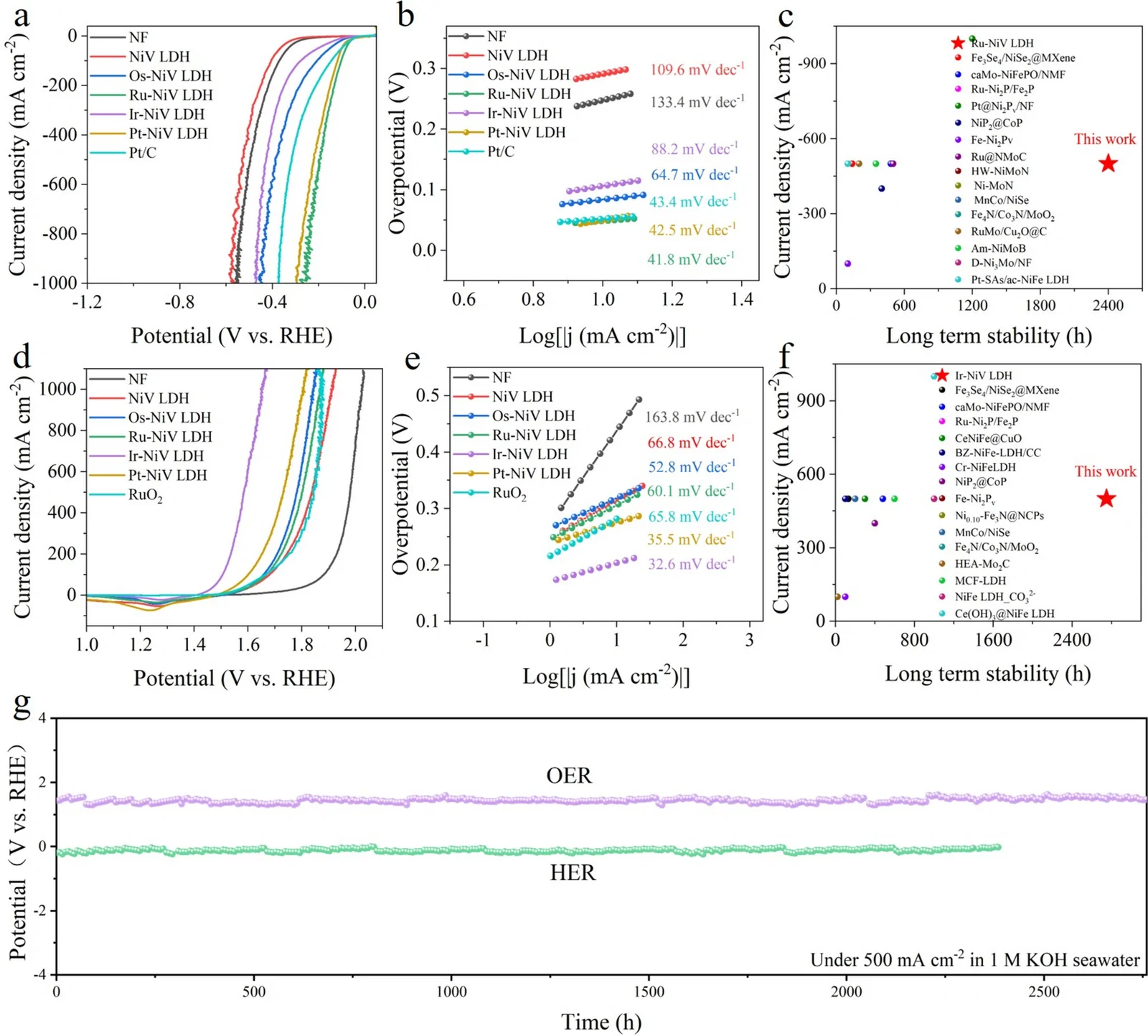
Electrochemical performance in 1 M KOH seawater. **a** HER polarization curve with iR compensation, **b** HER Tafel curve obtained from iR compensated polarization curve, **c** comparison with recently reported HER catalyst long-term stability and current density, **d** OER polarization curve with iR compensation, **e** OER Tafel curve obtained from iR compensated polarization curve, **f** comparison with recently reported OER catalyst long-term stability and current density, **g** chronopotentiometry curve of Ru-NiV LDH and Ir-NiV LDH at 500 mA cm^−2^

The hydrogen evolution reaction (HER) catalytic performance of the synthesized samples was evaluated using natural seawater from the South China Sea, supplemented with 1 M KOH to create an alkaline seawater electrolyte (Fig. S12). The iR-compensated polarization curves for nickel foam (NF), NiV LDH, Os-NiV LDH, Ru-NiV LDH, Ir-NiV LDH, Pt-NiV LDH, and commercial Pt/C were obtained (Fig. 2a). Among them, Ru-NiV LDH exhibited the highest HER catalytic activity. At high current densities of 500 mA cm^−2^, the overpotentials required for Ru-NiV LDH were 195 mV (Fig. S13a), significantly outperforming commercial Pt/C with 316 mV at the same current densities, a reduction of 121 mV in overpotential. To further investigate the HER kinetics of the catalysts, Tafel slopes were derived from the iR-compensated polarization curves. The Tafel slope of Ru-NiV LDH was 41.8 mV dec^−1^ (Fig. 2b), significantly lower than that of undoped NiV LDH (109.6 mV dec^−1^) and commercial Pt/C (43.4 mV dec^−1^), indicating that Ru doping substantially accelerated the HER kinetics. Additionally, electrochemical impedance spectroscopy (EIS) was conducted to analyze the charge transfer resistance (R_ct_) of the catalysts (Fig. S13b). According to the equivalent circuit model, Ru-NiV LDH exhibited the lowest charge transfer resistance (R_ct_ = 1.50 Ω), confirming its highly efficient electron transfer capabilities (Fig. S13b) and faster electrode reaction kinetics. The electrochemical surface area (ECSA) of the catalysts was evaluated using the double-layer capacitance (C_dl_) obtained from cyclic voltammetry (CV) measurements (Fig. S13c-i). The C_dl_ of Ru-NiV LDH was 8.63 mF cm^−2^, significantly higher than that of NiV LDH (0.41 mF cm^−2^) and NF (0.53 mF cm^−2^), indicating that Ru doping substantially increased the electrochemical surface area and exposed more active sites. Although Ru-NiV LDH exhibited a lower C_dl_ than Pt-NiV LDH (23.28 mF cm^−2^), this is likely due to fewer active sites counterbalanced by enhanced intrinsic activity, culminating in superior overall performance. At an industrial current density of 500 mA cm^−2^, Ru-NiV LDH demonstrated exceptional stability over 2350 h HER test (Fig. 2g), with a minimal decay rate of 0.072 mV h^−1^. The potential fluctuations during the test may have been caused by factors such as electrolyte replenishment, temperature variations, or bubble disturbances. Notably, most advanced HER electrocatalysts struggle to maintain long-term stability at current densities exceeding 500 mA cm^−2^, typically failing to operate continuously under such high current densities for more than 1000 h (Fig. 2c and Table S3). Furthermore, after 15,000 CV cycles (Fig. S14), the catalyst’s performance showed almost no change, further confirming its exceptional stability during the HER process. Post-stability testing, the surface morphology of Ru-NiV LDH retained its original layered structure (Fig. S15), indicating excellent structural stability. XRD patterns revealed that the crystal structure of Ru-NiV LDH remained intact without significant changes (Fig. S16). Additionally, XPS analysis revealed shifts in the binding energy of Ni 2*p*, V 2*p*, O 1*s*, and Ru 3*p* by 0.4, 0.6, 1.2, and 0.9 eV (Figs. S17 and S18), respectively, suggesting partial surface reduction during the HER process. Raman spectroscopy revealed no new peaks (Fig. S19), further confirming the structural stability of the catalyst. These results fully demonstrate the outstanding stability of Ru-NiV LDH in HER applications and its significant potential as a high-performance catalyst. Additionally, faradaic efficiency (FE) tests were further conducted on the Ru-NiV LDH electrode, revealing nearly 100% FE for H_2_ production. This result demonstrates that Ru doping effectively enhances the HER selectivity of the catalyst (Fig. S20).

Ru-NiV LDH exhibits the best HER catalytic performance, while Ir-NiV LDH demonstrates outstanding OER activity in 1 M KOH seawater (Fig. 2d). The iR-compensated OER polarization curves for NF, NiV LDH, Os-NiV LDH, Ru-NiV LDH, Ir-NiV LDH, Pt-NiV LDH, and commercial RuO_2_ are investigated. Ir-NiV LDH achieves an overpotential of only 202 mV at a current density of 10 mA cm^-2^, significantly lower than that of commercial RuO_2_ (286 mV). At an industrial-level current density of 500 mA cm^−2^, the Ir-NiV LDH catalyst exhibits an overpotential of 357 mV (Fig. S21a), significantly lower than that of commercial RuO_2_ (614 mV). Notably, a redox peak at 1.23 V was observed during the reverse scan of the OER polarization curve, corresponding to the anodic reduction reaction from Ni^3+^ to Ni^2+^. Considering the dynamic reconstruction of the catalyst surface structure before water oxidation, studying the redox electrochemical behavior of metal species in different catalysts is crucial, as it may have a decisive impact on the formation of active sites. Tafel analysis further demonstrates a marked improvement in kinetics (Fig. 2e), with Ir-NiV LDH exhibiting a Tafel slop of 32.6 mV dec^−1^, compared to 66.8 mV dec^−1^ for NiV LDH, indicating that Ir incorporation optimizes the electronic structure and accelerates the OER process. The Nyquist plot reveals that the Ir-NiV LDH electrode exhibits the smallest charge-transfer resistance among the tested catalysts, indicating highly efficient charge transfer and accelerated electrode reaction kinetics (Fig. S21b). Additionally, the C_dl_ of Ir-NiV LDH is 4.71 mF cm^−2^, higher than that of NF (0.53 mF cm^−2^) and NiV LDH (0.29 mF cm^−2^), indicating a larger electrochemical active surface area and exposure of abundant OER active sites (Fig. S21c-i). The layered structure of Ir-Ni V LDH enhances the electrocatalytic performance by both exposing abundant accessible active sites and promoting efficient mass/electron transport. Furthermore, the rapid detachment of gas bubbles reinforces mass transfer, which significantly accelerates the reaction kinetics, especially under high current densities relevant to industrial applications. These improvements can be attributed to the synergistic effects of morphology engineering and Ir doping, which collectively optimize the electronic structure of the catalyst. The long-term stability of the anode is critical for 1 M KOH seawater electrolysis, given the corrosive effects of ClO^−^ and Cl^−^ ions. In this study, we evaluated the electrochemical durability of Ir-NiV LDH using chronopotentiometry and accelerated degradation tests. After 15,000 CV cycles (Fig. S22), the polarization curve of Ir-NiV LDH exhibited minimal deviation, with performance showing virtually no degradation. Notably, at an industrial current density of 500 mA cm^−2^, the catalyst maintained stable OER activity for 2750 h (Fig. 2g), with only slight performance attenuation (0.011 mV h^−1^), despite potential fluctuations likely due to electrolyte replenishment, temperature variations, and bubble disturbances. By comparison, most advanced OER electrocatalysts can operate continuously for less than 1000 h at similar current densities (Fig. 2f and Table S4). Post-stability characterizations further confirmed the catalyst’s robustness: SEM images showed that the layered morphology remained largely unchanged (Fig. S23), while XRD patterns confirmed that the crystal structure was intact (Fig. S24). XPS analysis indicated an increase in the oxygen vacancy peak in the O 1*s* spectrum after stability testing (Figs. S25 and S26), suggesting that the formation of additional oxygen vacancies may further enhance OER activity. In summary, these results demonstrate that Ir-NiV LDH exhibits exceptional OER activity and long-term stability in seawater electrolysis, underscoring its significant potential for industrial water splitting applications.

### Electrocatalytic Mechanism

**Fig. 3:**
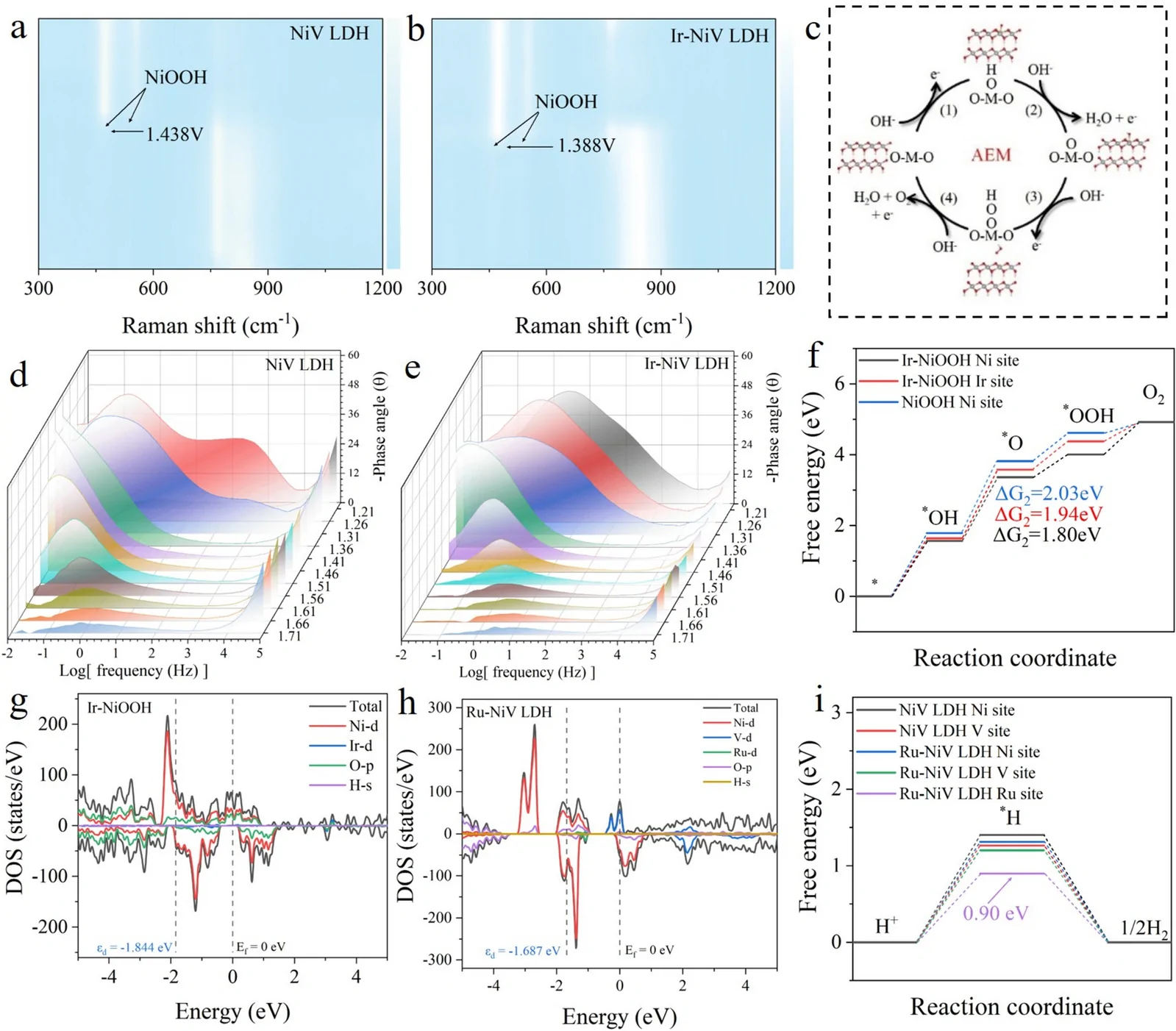
Catalytic mechanism. **a** In situ Raman contour maps of NiV LDH, **b** In situ Raman contour maps of Ir-NiV LDH, **c** AEM mechanism, **d** Bode plots of NiV LDH at different potentials, **e** Bode plots of Ir-NiVLDH at different potentials, **f** free energy diagram for OER on NiOOH and Ir-NiOOH, **g** projected density of states of Ir-NiV LDH, **h** projected density of states of Ru-NiV LDH, **i** free energy diagram for HER on NiV LDH and Ru-NiV LDH

To provide a comprehensive understanding of the enhanced catalytic activity observed in Ir-NiV LDH, we employed a multifaceted approach encompassing in situ Raman spectroscopy, potential-dependent Bode plots, and theoretical calculations. In situ Raman spectroscopy was pivotal in elucidating the origin of the improved OER performance. For NiV LDH in 1 M KOH, the emergence of new Raman peaks at 474 and 554 cm^−1^ was observed at an anodic potential of 1.438 V (Fig. 3a), which are assigned to the NiOOH phase [38]. In the OER process, NiOOH is generally recognized as the true catalytically active species, and earlier reconstruction of NiOOH correlates with better catalytic activity. In contrast, the characteristic NiOOH peak for Ir-NiV LDH appears at a lower potential of 1.388 V indicating that Ir incorporation facilitates the Ni(OH)_2_ to NiOOH phase transition (Figs. 3b and S27), concomitant with the oxidation of Ni^2+^ to Ni^3+^. Operando EIS further revealed insights into interfacial dynamics and electron transfer mechanisms during OER. The Bode plots for both NiV LDH and Ir-NiV LDH demonstrated notable changes with increasing applied potential (Fig. 3d, e) [35] Specifically, at 1.56 V, the phase angle in the low-frequency region (10^-2^-10 Hz) represents the OER interface, while the high-frequency region (10-10^5^ Hz) is related to the surface oxidation process. The phase angle decreases in the low-frequency region. At a voltage of 1.56 V, the phase angle of NiV LDH is about 16°, while that of Ir-NiV LDH is about 6°, indicating that the OER rate at the interface of the latter is faster [39]. Simultaneously, the Bode plot exhibits a peak in the low-frequency region, which shifts to a lower frequency as the potential increases. This can be attributed to the adsorption of OH* intermediates. The phase angle in the low-frequency region decreases sharply. The voltage for NiV LDH is 1.46 V, while for Ir-NiV LDH, it is 1.41 V, reflecting that Ir-NiV LDH has a faster OER rate, consistent with the in situ Raman spectroscopy and polarization curves. DFT calculations provided deeper insight into the electronic structure modifications induced by Ir doping. The actual OER active species NiOOH and Ir-NiOOH were selected as the calculation model (Fig. S35). Density of states (DOS) analysis revealed an upward shift in the DOS of Ir-NiOOH relative to the Fermi level compared to NiOOH (Figs. 3g and S34a), facilitating electron removal from oxygen sites and reducing the energy absorption barrier, thereby enhancing OER activity [40]. Furthermore, an in-depth examination of the surface-reconstructed Ir-NiOOH indicated that the catalytic active sites in both Ir-NiOOH and pristine NiOOH are exposed Ni centers, with intermediates (*OH, *O, and *OOH) being continuously adsorbed. At U = 0, the activation energy barrier from *OH to *O is 2.03 eV, constituting the rate-determining step (RDS) of the reaction (Figs. S40-42). Notably, Ir doping reduced this barrier to 1.80 eV (Fig. 3f), suggesting that Ir incorporation effectively modulates the electronic structure of Ni sites, enhancing their OH adsorption capacity [38, 41]. Collectively, these findings demonstrate that Ir-NiV LDH significantly enhances OER performance by optimizing the electronic structure and strengthening intermediate adsorption capabilities (Fig. 3c), offering valuable theoretical insights for its application in seawater electrolysis. To further elucidate the OER mechanism, we conducted differential electrochemical mass spectrometry (DEMS) with isotopic labeling. The ^32^O_2_ to ^34^O_2_ signal ratios for ^18^O-labeled Ir-NiV LDH and NiV LDH were 208.5 and 191.5, respectively, indicating that the introduction of Ir significantly enhances the intramolecular O-O coupling pathway, consistent with the adsorbate evolution mechanism (AEM) (Fig. S43). Moreover, although Ir-NiV LDH exhibited higher total oxygen evolution, the proportion of the side product ^18^O^16^O (^34^O_2_) in the total O_2_ (0.48%) was lower than that of NiV LDH (0.52%), further confirming the high stability of the lattice oxygen in these catalysts and its negligible participation in the oxidation reaction. These results support the conclusion that the AEM pathway predominates and reinforce the understanding that Ir modification promotes the AEM route.

In addition to explaining the OER mechanism, we also provided an explanation of the HER mechanism. DFT calculations were performed on NiV LDH and Ru-NiV LDH models (Fig. S35). The calculated DOS shows that Ru-NiV LDH has a DOS (-1.687 eV) closer to the Fermi level (0 eV) compared to NiV LDH (−1.713 eV) (Figs. 3h and S34b) [42], indicating enhanced electronic conductivity due to Ru incorporation. This modulation of the d-band center optimizes hydrogen intermediate adsorption, lowering the energy barriers for both the Tafel rate-determining step and hydrogen migration. The hydrogen adsorption Gibbs free energy (G_H*_) for Ru-NiV LDH is 0.90 eV (Fig. 3i), significantly lower than that of NiV LDH (1.40 eV), establishing Ru-NiV LDH as the most efficient HER active center (Figs. S38 and S39). These theoretical results confirm that Ru doping effectively enhances the intrinsic HER kinetics by fine-tuning the catalyst’s electronic structure. In summary, the optimized electronic structure and enhanced intrinsic activity induced by Ru doping underscore the importance of tailored catalyst design for efficient and stable HER performance in seawater electrolysis. These findings highlight the potential of Ru-NiV LDH as a high-performance catalyst for hydrogen evolution. In addition, the surface water contact angles of the catalysts were measured to evaluate their hydrophilicity and water capture capability. Upon contact, water droplets exhibited instantaneous spreading and complete absorption on all three samples. Notably, Ir-NiV LDH and Ru-NiV LDH demonstrated a significantly shorter time for complete droplet penetration compared to NiV LDH, indicating their superior water capture capacity and enhanced hydrophilicity (Fig. S33).

### Dual Chloride Confinement Mechanism

**Fig. 4:**
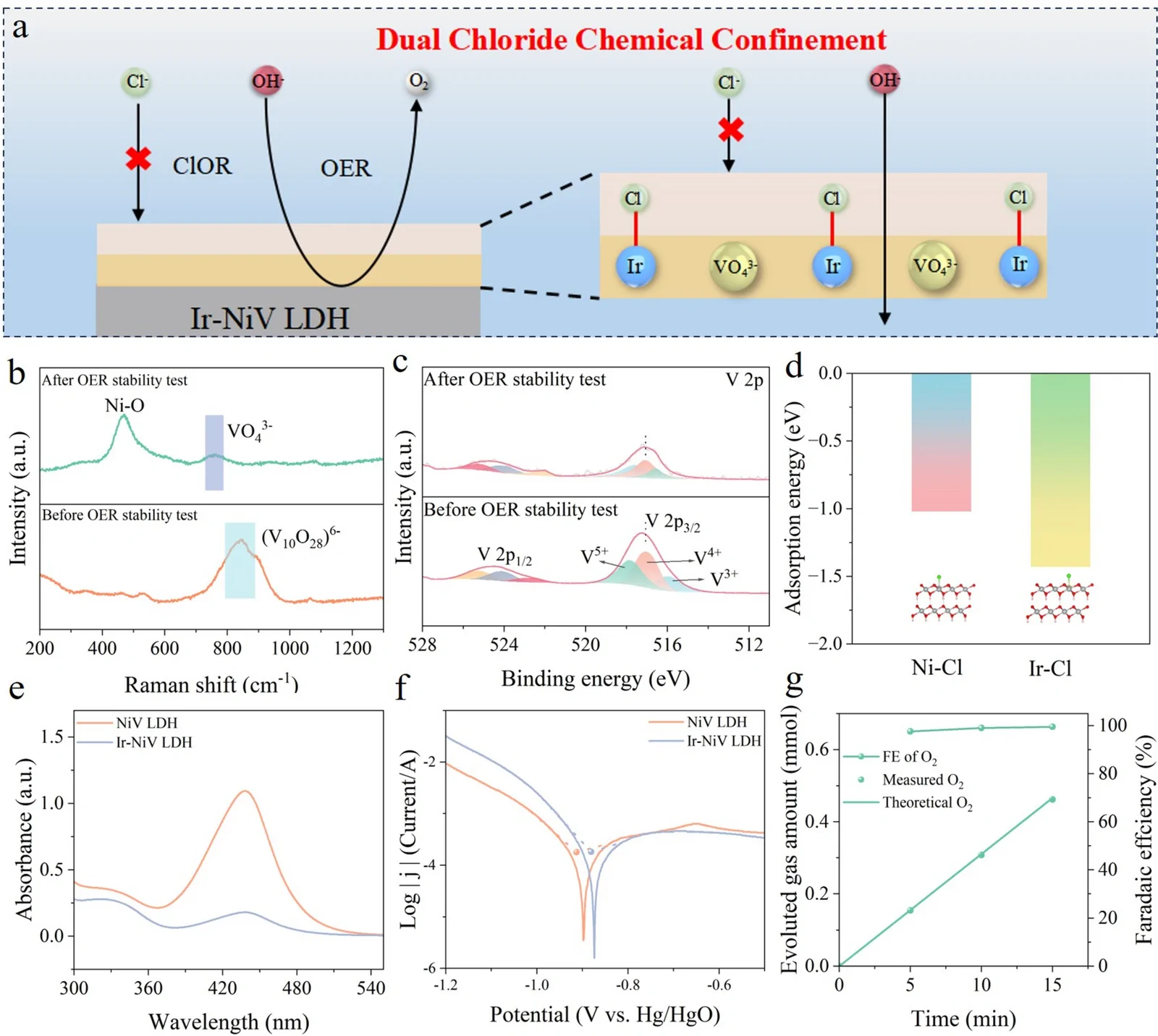
**a** Schematic diagram of Ir-NiV LDH in the process of seawater oxygen evolution and the mechanism of Cl^-^ rejection, **b** Raman spectra of Ir-NiV LDH before and after stability test, **c** XPS of V 2*p* of Ir-NiV LDH before and after stability test, **d** Cl adsorption energy of NiOOH and Ir-NiOOH, **e** UV-VIS spectra of NiV LDH and Ir-NiV LDH were tested by o-tolidine, **f** Tafel polarization curves for NiV LDH and Ir-NiV LDH, **g** experimental and theoretical gaseous products (O_2_) by the two-electrode electrolyzer

The presence of Cl^-^ in seawater triggers the competing ClOR, which significantly suppresses the OER by corroding catalyst surfaces. To address this challenge, we designed a dual chlorine chemical confinement layer catalyst. This architecture comprises in situ formation of VO_4_^3−^ layer and an Ir-Cl adsorption layer (Fig. 4a), which synergistically prevents Cl^−^ induced corrosion through electrostatic repulsion. The presence of VO_4_^3−^ in the Ir-NiV LDH catalyst was confirmed by Raman spectroscopy. Raman spectroscopy was employed to investigate the structural changes of the Ir-NiV LDH catalyst before and after the stability test. In the initial spectrum, aside from the two characteristic peaks attributable to Ni-O vibrations, an additional peak was observed at 843 cm^−1^. This observed peak is attributed to the symmetric V-O-V stretching vibration of the (V_10_O_28_)^6−^ species (Fig. 4b) [7, 43, 44]. After stability testing, we observed the disappearance of this peak and the emergence of a new peak at 760 cm^-1^, corresponding to VO_4_^3-^ [7]. The spectral evolution indicates the operational dissolution of V species, accompanied by a transformation of the Ir-NiV LDH from(V_10_O_28_)^6-^ to VO_4_^3-^. We propose that under the applied anodic potential, dissolved V species are maybe re-adsorbed onto the electrode surface, reforming a protective VO_4_^3−^ layer. This dynamic regeneration process effectively creates an electrostatic barrier that repels Cl^−^, thereby enhancing the catalyst’s stability in chloride-containing electrolytes. Furthermore, XPS analysis provided additional evidence supporting this conclusion. V 2*p* spectra exhibited a significant decrease in peak intensity along with a noticeable binding energy shift (Fig. 4c), consistent with the dissolution of V species [7]. After the stability test, the relative peak area of V^3+^ markedly decreased, whereas those of V^4+^ and V^5+^ increased. This systematic shift toward higher oxidation states provides crucial evidence for the valence evolution associated with the structural transformation from (V_10_O_28_)^6-^ to VO_4_^3−^. Together with the Raman spectroscopy results, these spectroscopic observations collectively suggest the possible formation of a V^5+^-enriched protective layer on the electrode surface under testing conditions, with VO_4_^3-^. likely being its primary constituent.

Further evidence for the formation of Ir-Cl species was provided by in situ Raman spectroscopy. When measured in 1 M KOH + 0.5 M NaCl electrolyte, a weak but discernible peak was observed at 350 cm^−1^, which corresponds to the characteristic vibration of Ir-Cl bonding (Fig. S29) [26]. Additionally, DFT calculations were employed to investigate Cl adsorption behavior. The computational results demonstrate that Ir sites exhibit significantly enhanced chloride adsorption capability, with a more negative adsorption energy (−1.51 eV) compared to Ni sites (−1.02 eV) (Figs. 4d and S37) [9] This substantial energetic difference confirms the formation of an Ir-Cl protective layer through preferential chloride chemisorption [9]. Remarkably, the strong Ir-Cl interaction establishes an electrostatic screening effect that effectively prevents chloride-induced corrosion of adjacent Ni active sites. The results demonstrate that the catalyst exhibits excellent OER catalytic activity and enhanced stability. Complementary characterization techniques including UV-vis spectroscopy, corrosion current measurements, and Faradaic efficiency analysis were employed to further verify the dual chloride confinement mechanism [38]. Systematic UV-vis spectral monitoring under industrial current densities revealed significantly lower concentrations of active chlorine species in the electrolyte for Ir-NiV LDH compared to the NiV LDH control (Fig. 4e), demonstrating effective suppression of Cl^-^ oxidation (Fig. S31). Furthermore, i-t tests conducted in a perchlorate-containing electrolyte revealed that Ir-NiVLDH exhibits enhanced structural stability compared to NiV LDH, along with a reduced incidence of ClOR. These findings further substantiate the chloride-repelling mechanism inherent to Ir-NiV LDH (Fig. S32). The corrosion assessments through polarization curves showed that Ir-NiV LDH exhibits both higher corrosion potential and lower corrosion current density than NiV LDH (Fig. 4f), quantitatively confirming superior chloride corrosion resistance. These results provide direct experimental evidence that the in situ formation of VO_4_^3−^ layer and strong Ir-Cl adsorption effectively protects electrode integrity from Cl^-^ attack. Additionally, FE tests were further conducted on the Ir-NiV LDH electrode, revealing nearly 100% FE for O_2_ production (Figs. 4g and S30), demonstrating exceptional OER selectivity. The synergistic combination of these experimental results and theoretical calculations finally confirmed the design of dual chlorine chemical confinement layer. This dual chlorine chemical confinement highlights the great potential of Ir-NiV LDH for practical industrial seawater electrolysis applications, addressing both stability and selectivity challenges in seawater electrolytes.

**Fig. 5:**
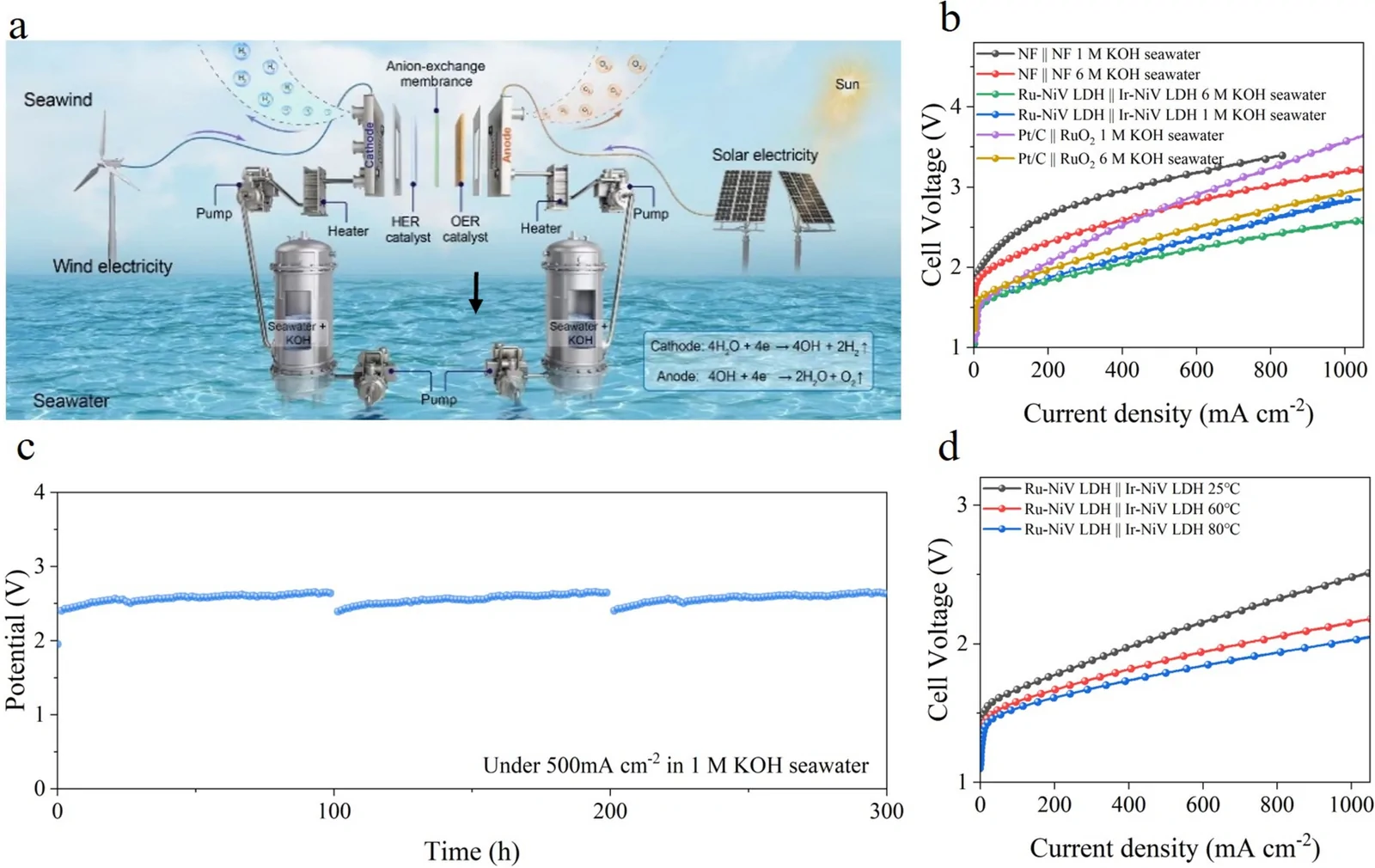
Commercial AEM performance (without iR compensation) in alkaline seawater electrolyte. **a** Schematic diagram of AEM electrolyzer driven by green energy (wind energy, solar energy) in the future is proposed, **b** polarization curves of the AEM electrolyzer using Ru-NiV LDH || Ir-NiV LDH, NF || NF and Pt/C || RuO_2_, **c** durability test at a constant current density of 500 mA cm^−2^, **d** polarization curves of the Ru-NiV LDH || Ir-NiV LDH measured in 6 M KOH + seawater at different temperatures

Benefiting from the excellent HER and OER catalytic performance of Ru-NiV LDH and Ir-NiV LDH, we investigated the overall seawater electrolysis performance using a two-electrode system (electrode area: 1 cm^2^) and a commercial electrolyzer (electrode area: 4 cm^2^). A two-electrode system was constructed with commercial Ru-NiV LDH as the cathode and Ir-NiV LDH as the anode (Ru-NiV LDH || Ir-NiV LDH) (Fig. S28a). Ru-NiV LDH || Ir-NiV LDH exhibited outstanding activity, achieving a current density of 500 mA cm^−2^ at a cell voltage of only 2.98 V, outperforming NF || NF (3.60 V). Furthermore, the long-term stability of Ru-NiV LDH || Ir-NiV LDH was evaluated over 100 h of seawater electrolysis at an industrial current density of 500 mA cm^−2^ (Fig. S28b), with only negligible potential decay observed, demonstrating its excellent stability. To further assess its practical application potential, a mode of AEM electrolytic cell driven by green energy (wind energy, solar energy) in the future is proposed (Fig. 5a). This commercial electrolyzer was assembled using Ru-NiV LDH and Ir-NiV LDH electrodes. The polarization curve of Ru-NiV LDH || Ir-NiV LDH (without iR compensation) displayed excellent catalytic performance (Fig. 5b). At 500 mA cm^−2^, the overall water splitting voltages of the Ru-NiV LDH || Ir-NiV LDH electrode in 1 M KOH seawater and 6 M KOH seawater were 2.24 and 2.14 V, respectively. These values are significantly lower than those of the NF electrode (3.07 and 2.71 V) and the Pt/C || RuO_2_ reference electrode (2.72 and 2.38 V), demonstrating its superior catalytic activity. Additionally, the commercial electrolyzer using Ru-NiV LDH || Ir-NiV LDH operated stably for over 300 h at a current density of 500 mA cm^−2^ (Fig. 5c), with no significant failure observed during seawater electrolysis, demonstrating its potential commercial value. It is worth noting that its performance was slightly lower than that of the two-electrode system (Fig. S28b). This discrepancy can be mainly attributed to mass transport limitations in the scaled electrode and device-related structural factors-including flow field design and membrane selection-which collectively influence catalytic performance. The interruptions observed during the stability tests resulted from periodic start-stop cycles to simulate intermittent real operation, and electrolyte replacement to prevent cumulative concentration deviations. Nevertheless, these results indicate that Ru-NiV LDH and Ir-NiV LDH are outstanding seawater electrolysis catalysts. The strong stability observed in both the two-electrode system and the commercial electrolyzer is attributed to the dual chemical confined layer designed in this study, achieved through the generation of VO_4_^3-^ and the strong electrostatic adsorption of Ir-Cl. The enhanced catalytic performance confirms the potential of this catalyst for industrial seawater electrolysis. The AEM cell efficiency were calculated [25]. The electrolyzer efficiency of the assembled device at 100 mA cm^−2^ in 6 M KOH seawater was 73.9%. Additionally, polarization curves of Ru-NiV LDH and Ir-NiV LDH were evaluated under simulated industrial conditions (6 M KOH + seawater) at various temperatures. The results indicate that the catalysts exhibit significantly enhanced activity at 80 °C, achieving a current density of 500 mA cm^−2^ at a cell voltage of only 1.79 V (Fig. 5d). These results demonstrate that the Ru-NiV LDH || Ir-NiV LDH catalyst not only exhibits excellent catalytic activity and stability, but also offers significant economic advantages, providing strong support for its widespread application in industrial seawater electrolysis.

## Conclusions

In summary, we have demonstrated a one-step synthesis strategy for noble metal-doped NiV LDH catalysts that effectively address the key challenges of direct seawater electrolysis. By harnessing precise electronic modulation and establishing a dual chemical confinement mechanism, via strong Ir-Cl adsorption combined with the in situ generation of protective VO_4_^3−^ layers, we have achieved catalysts that maintain high activity and robust durability under the aggressive, chloride-rich conditions of seawater. Our optimized Ru‐doped and Ir‐doped NiV LDH catalysts exhibit exceptional performance, with HER and OER overpotentials as low as 195 and 357 mV, respectively, at an industrially relevant current density of 500 mA cm^−2^, and operational stabilities exceeding 2350 and 2750 h. Moreover, when integrated into commercial electrolyzers, this approach enables efficient hydrogen production at a lower operating voltage, thereby demonstrating promising potential for industrial application. Collectively, these advances not only deepen our understanding of catalyst behavior in complex ionic environments, but also lay a viable foundation for scalable, cost-effective green hydrogen production from seawater.

## Supplementary Information

Below is the link to the electronic supplementary material.

Supplementary file1 (DOCX 15533 KB)
