# Supplementary material for: Dual Chloride Confinement in Noble Metal‐Doped NiV LDH Catalysts Enables Stable Industrial-Level Seawater Electrolysis
Source: Nanomicro Lett. 2026 Jan 16;18:210. doi: 10.1007/s40820-026-02067-1 (PMC12811202; doi:10.1007/s40820-026-02067-1)
Supplement: Supplementary file 1 — Supplementary file1 (DOCX 15533 KB) [file 40820_2026_2067_MOESM1_ESM.docx]

Supporting Information for

**Dual Chloride Confinement in Noble Metal‐Doped NiV LDH Catalysts Enables Stable Industrial-Level Seawater Electrolysis**

Kai Liu^1^, Yaohai Cai^1^, Xiaotian Wei^1^, Lihang Qu^1^, Jianxi Lu^1^, Yingwei Qi^1^, Zhenbo Wang^2,*^, Dong Liu^1,*^

^1^ Guangdong Provincial Key Laboratory of New Energy Materials Service Safety, College of Materials Science and Engineering, Shenzhen University, Shenzhen 518060, P. R. China

^2^ State Key Laboratory of Space Power-Sources, MIIT Key Laboratory of Critical Materials Technology for New Energy Conversion and Storage, MOE Engineering Research Center for Electrochemical Energy Storage and Carbon Neutrality in Cold Regions, School of Chemistry and Chemical Engineering, Harbin Institute of Technology, Harbin 150001, P. R. China

*Corresponding authors. E-mail: [dongliu@szu.edu.cn](mailto:dongliu@szu.edu.cn) (Dong Liu); [wangzhb@hit.edu.cn](mailto:wangzhb@hit.edu.cn) (Zhenbo Wang)

**S1 Experiment Sections**

**S1.1 Materials**

Nickel foam (NF), nickel nitrate (Ni(NO_3_)_2_·6H_2_O; purity ≥98%), vanadium chloride (VCl_3_; purity ≥98%), iridium chloride hydrate (IrCl_3_·xH_2_O; purity ≥98%), ruthenium chloride hydrate (RuCl_3_·xH_2_O; purity ≥98%), osmium chloride (OsCl_3_; purity ≥98%), chloroplatinic acid (H_2_PtCl_6_; purity ≥98%), urea (CN_2_H_4_O; purity ≥98%), potassium hydroxide (KOH; purity ≥98%), commercial RuO_2_, and commercial Pt/C (20%). Hydrochloric acid, acetone, absolute alcohol, and deionized water with a resistivity of 18.2 MΩ were utilized in all experiments. Additionally, seawater was sourced from the South China Sea (Shenzhen Bay).

**S1.2** **Electrochemical measurements**

Electrochemical evaluations of all samples were performed using CHI660E, CHI760E DH7006A-2, and DH7006B-2 stations in an alkaline seawater electrolyte, prepared by adding 1 M KOH to natural, unpurified seawater collected from the South China Sea. A Hg/HgO electrode and a graphite rod served as the reference and counter electrodes, respectively, for the oxygen evolution reaction (OER) and hydrogen evolution reaction (HER). All the prepared samples were utilized as working electrodes. Potentials in these experiments were adjusted to the reversible hydrogen electrode (RHE) standard using the formula: E_RHE_= E + 0.098 V + 0.059 × pH. The electrocatalytic performance was assessed in various electrolytes: 1.0 M KOH and 1.0 M KOH seawater solution. Linear sweep voltammetry (LSV) with a scan rate of 5 mV s^-1^ was employed to evaluate the OER and HER activities. Tafel slopes were determined from the linear regions of the Tafel plots using the Tafel equation upon LSV curves with iR compensation. Cyclic voltammetry (CV) tests were performed at varying scan rates (20, 40, 60, 80, and 100 mV s^-1^) to determine the double-layer capacitance (*C_dl_*) of the catalysts. Electrochemical impedance spectroscopy (EIS) was conducted across a frequency range of 0.01 kHz to 10000 kHz. Long-term stability was assessed via chronopotentiometry at room temperature. The synthesized sample, Ag/AgCl electrode and Pt wire were used as working electrode, reference electrode and counter electrode, respectively. The measurement was carried out in 1 M potassium hydroxide with a potential range from 0.1 V to 0.6 V (vs. Ag/AgCl). After 15 s of testing, data were collected.

**S1.3 Calculations of AEM electrolyzer efficiency** **(6 M KOH seawater)**

These calculations only considered the electricity costs, based on the method proposed by literature:

H_2_ production rate @ 0.1 A cm^-2^

= (j A cm^-2^) (1 e^-^/1.602 × 10^-19^ C) (1 H_2_/2 e^-^)

= 0.1 A cm^-2^ / (1.602 × 10^-19^ C × 2) = 5.1828 × 10^-7^ mol H_2_ cm^-2^ s^-1^

LHV of H_2_

= 120 kJ g^-1^ H_2_ = 2.42 × 10^5^ J mol^-1^ H_2_

H_2_ power out

= (5.1828 × 10^-7^ mol cm^-2^ s^-1^) × (2.42 × 10^5^ J mol^-1^) = 0.1254 W cm^-2^

Electrolyzer Power @ 0.1 A cm^-2^ (6 M KOH seawater)

= (0.1 A cm^-2^) (1.698V) = 0.1715 W cm^-2^

Efficiency of AEM

= (H_2_ Power Out) / (Electrolyzer Power) = 0.1254 W cm^-2^/ 0.1698W cm^-2^ = 73.9%

**S1.4 DFT calculation**

All the DFT calculations were conducted based on the Vienna Ab-inito Simulation Package (VASP). The exchange-correlation effects were described by the Perdew-Burke-Ernzerhof (PBE) functional within the generalized gradient approximation (GGA) method. The core-valence interactions were accounted by the projected augmented wave (PAW) method. The energy cutoff for plane wave expansions was set to 400 eV, and the 3×3×1 Monkhorst-Pack grid k-points were selected to sample the Brillouin zone integration. The structural optimization was completed for energy and force convergence set at 1.0×10^-4^ eV and 0.05 eV Å^-1^, respectively.

The adsorption energies (*E*_ads_) of Cl are calculated by:

*E*_ads_ = *E*_*Cl_–*E*_Cl_–*E*_Sub_

where *E*_Cl_ and *E*_*Cl_ represent the energies before and after the adsorption of Cl on the substrates, respectively. *E*_sub_ is the energy of NiOOH and Ir-NiOOH surfaces.

The Gibbs free energy change (ΔG) of each step is calculated using the following formula:

∆G = ∆E + ∆ZPE - T∆S

where ΔE is the electronic energy difference directly obtained from DFT calculations, ΔZPE is the zero point energy difference, T is the room temperature (298.15 K) and ΔS is the entropy change. ZPE could be obtained after frequency calculation by:

ZPE =$\frac{1}{2} \sum hvi$

And the TS values of adsorbed species are calculated according to the vibrational frequencies:

$$TS = k_{B}T [\sum_{k} ln(\frac{1}{1-e^{-hv/k_{B}T}})+ \sum_{k} \frac{hv}{k_{B}T} \frac{1}{{(e}^{hv/k_{B}T}-1)}+1]$$

**Supplementary Figures and Tables**


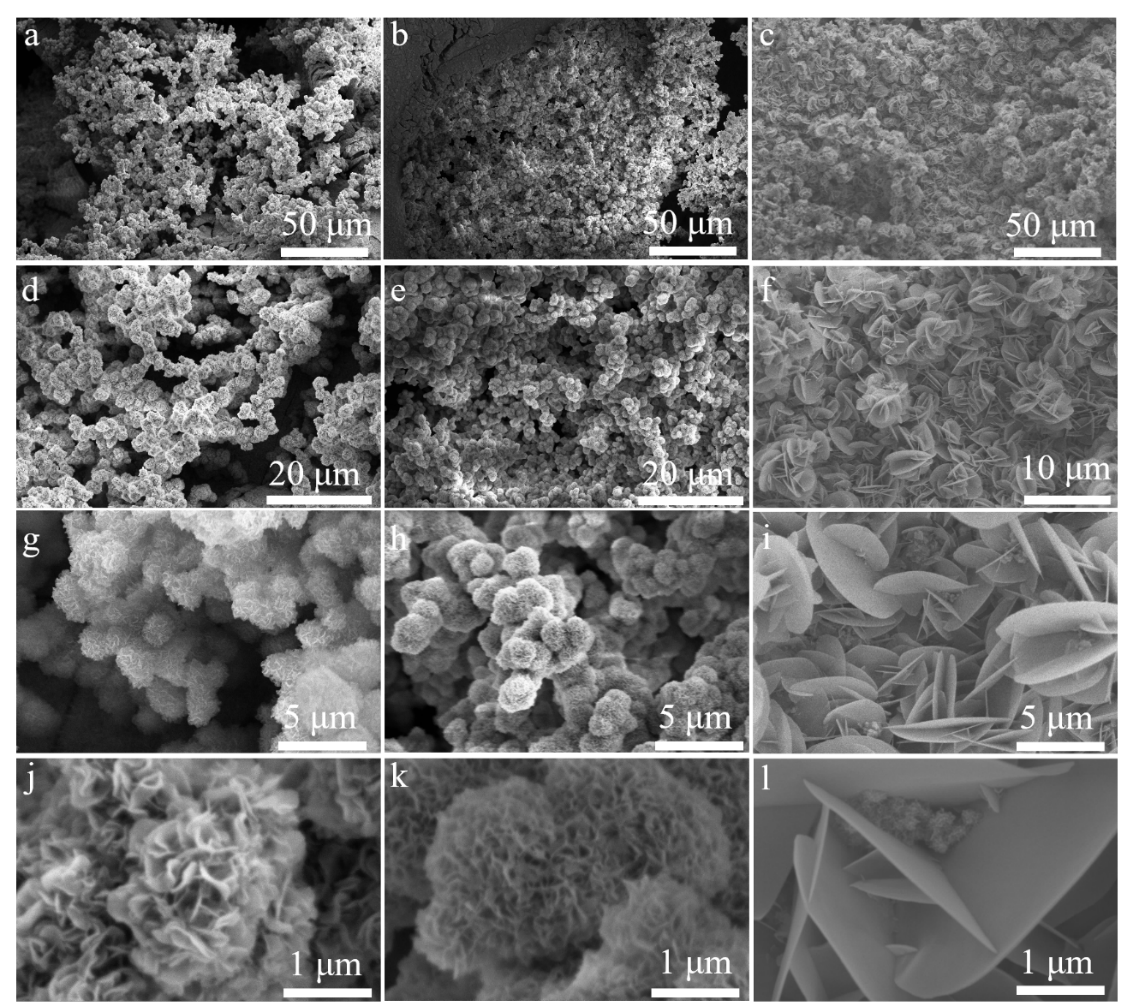


**Fig. S1** SEM images of (**a, d, g, j**) NiV LDH, (**b, e, h, k**) Ru-NiV LDH, and (**c, f, i, l**) Ir-NiV LDH at different magnifications. Scale bars: 50 μm (**a-c**), 20 μm (**d-f**), 5 μm (**g-i**), and 1 μm (**j-l**)


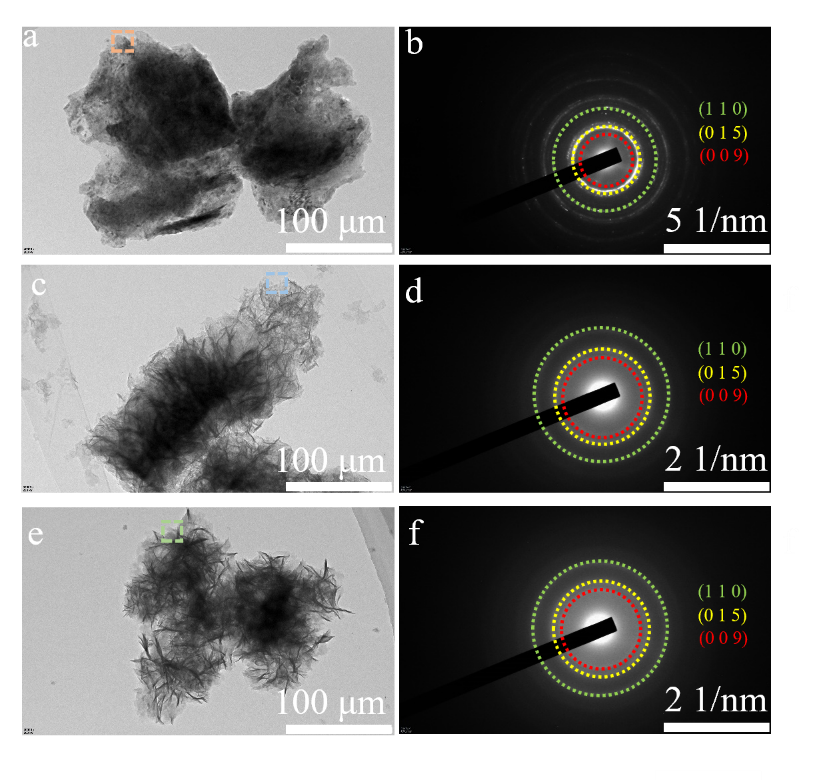


**Fig. S2** TEM images of **a**) NiV LDH, **c**) Ru-NiV LDH, and **e**) Ir-NiV LDH, SAED pattern of **b**) NiV LDH, **d**) Ru-NiV LDH, and **f**) Ir-NiV LDH


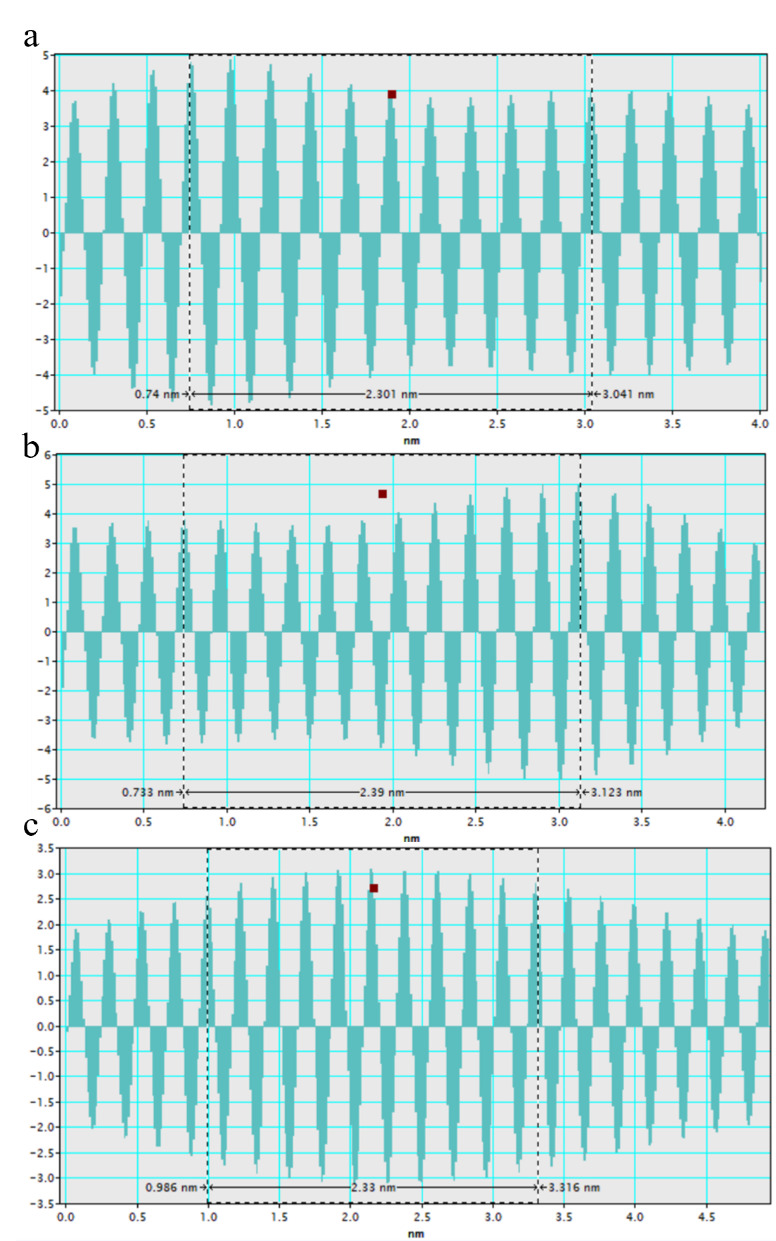


**Fig. S3 a-c**) Corresponding line intensity file taken from the selected areas of **Fig.** 1b,1c, and 1d, respectively


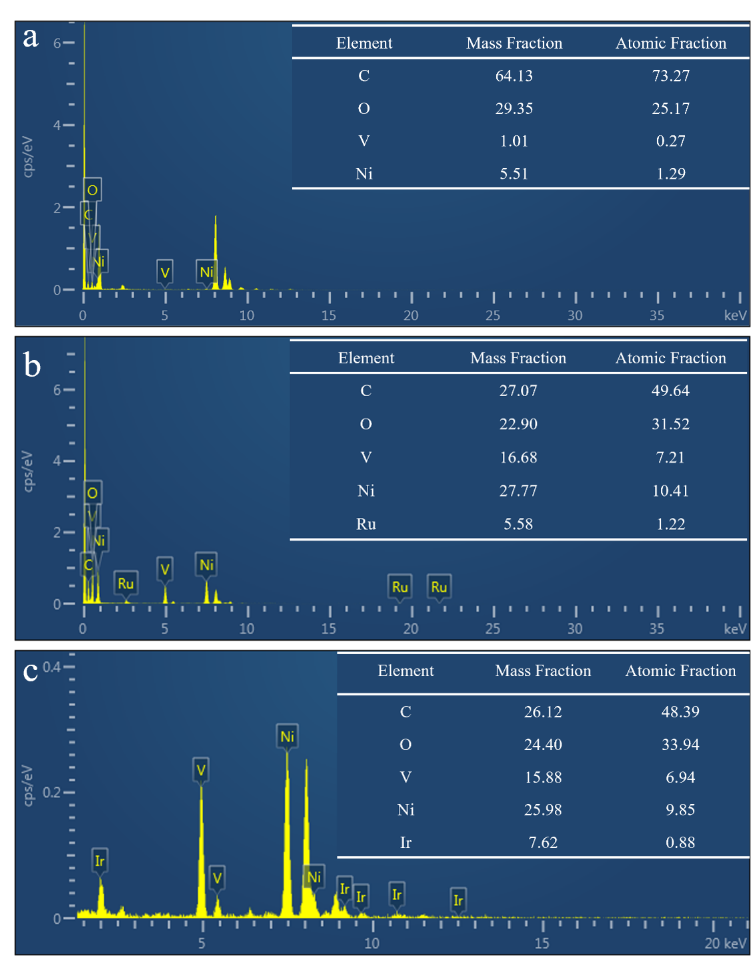


**Fig. S4** TEM-EDX results of **a**) NiV LDH, **b**) Ru-NiV LDH, and **c**) Ir-NiV LDH


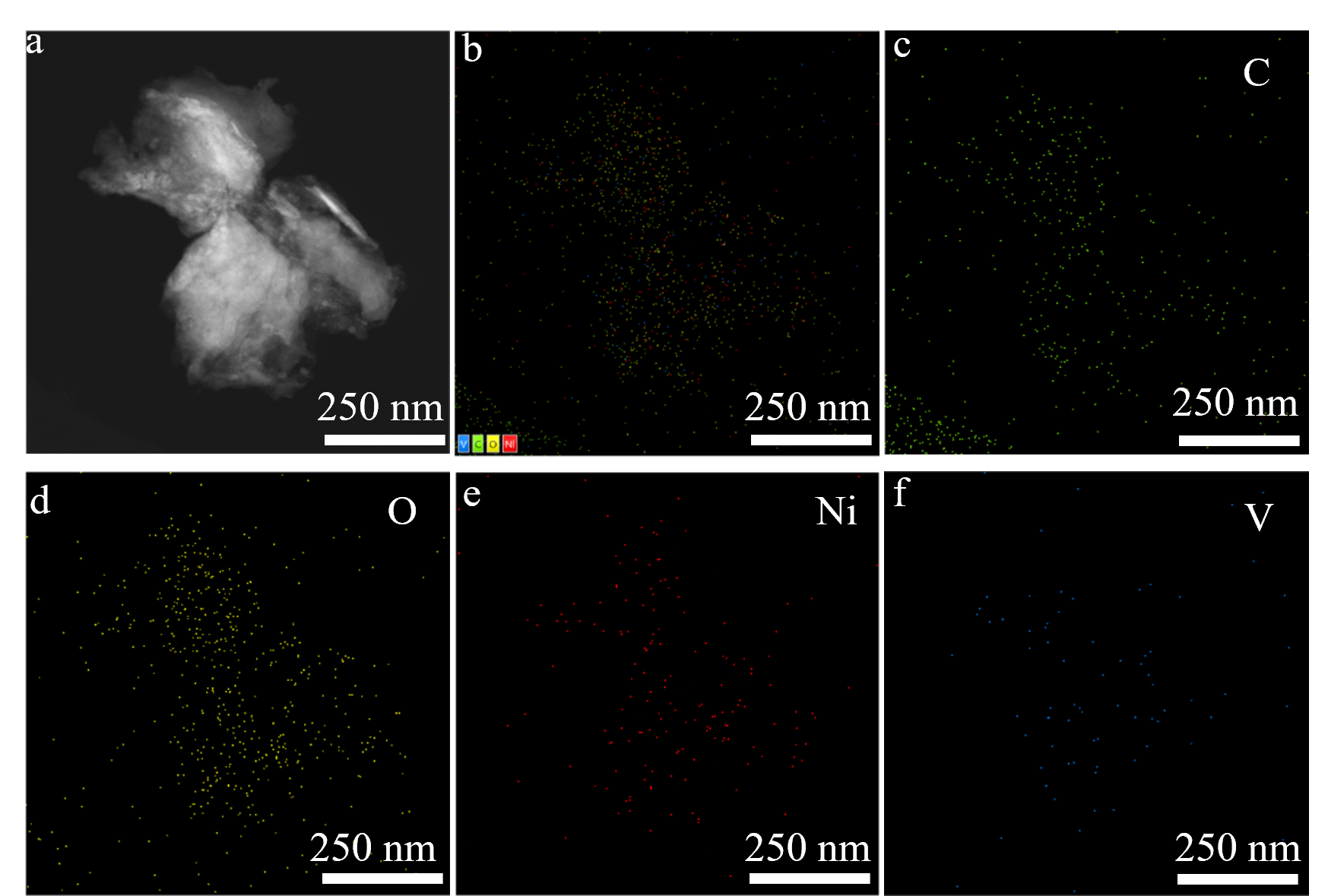


**Fig. S5** TEM image for NiV LDH with elemental mapping images for C, O, Ni, and V elements


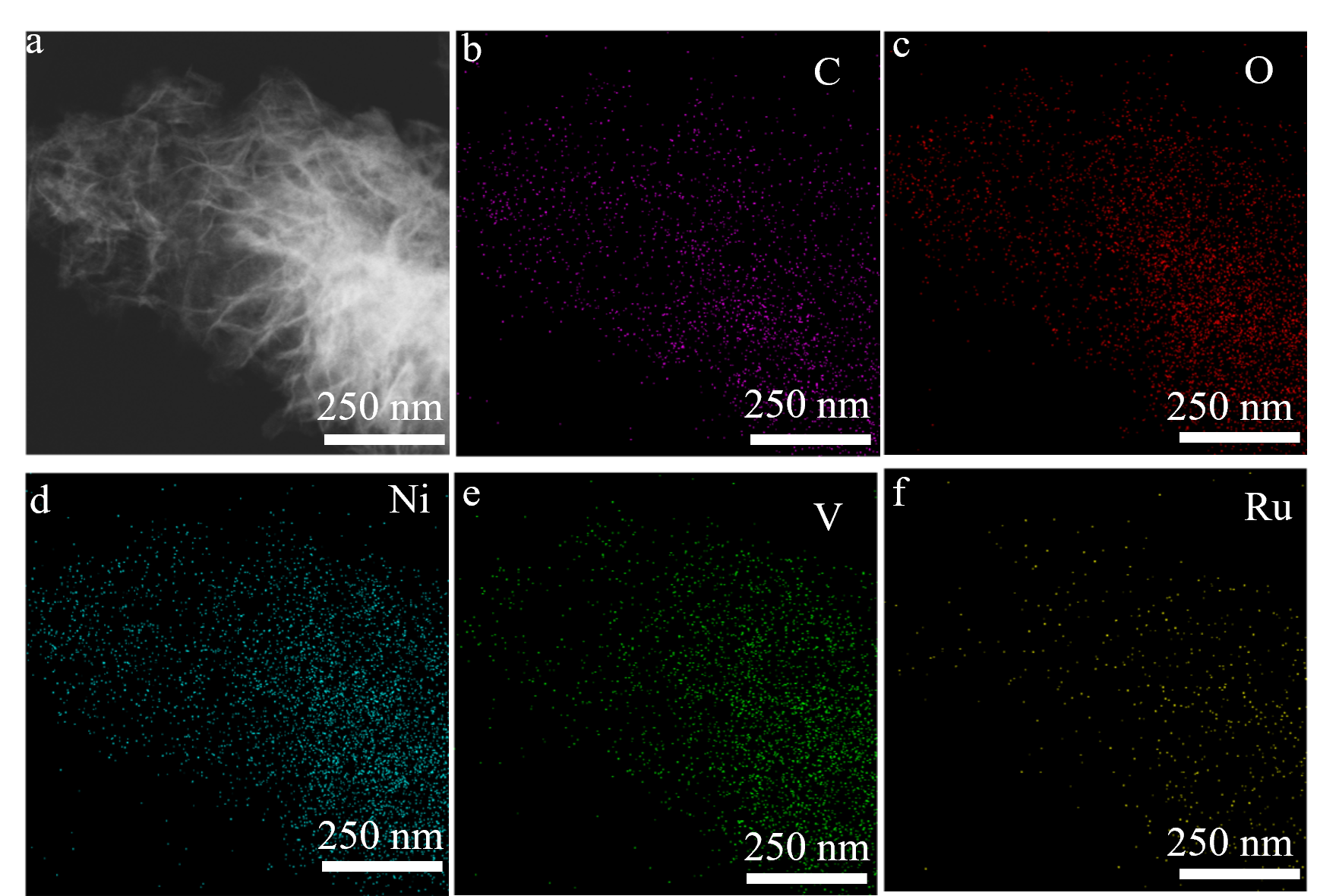


**Fig. S6** TEM image for Ru-NiV LDH with elemental mapping images for C, O, Ni, V, and Ru elements


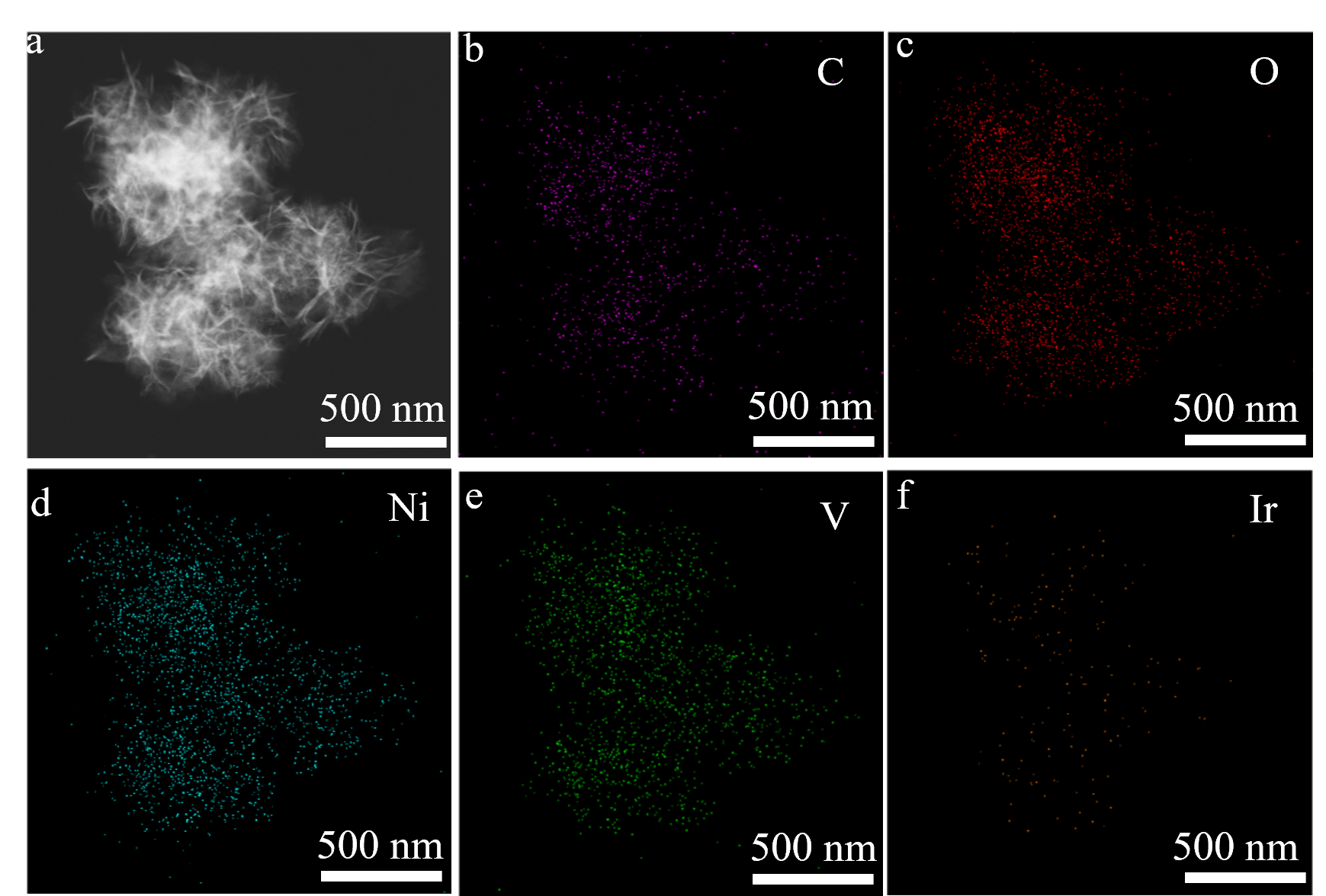


**Fig. S7** TEM image for Ir-NiV LDH with elemental mapping images for C, O, Ni, V, and Ir elements


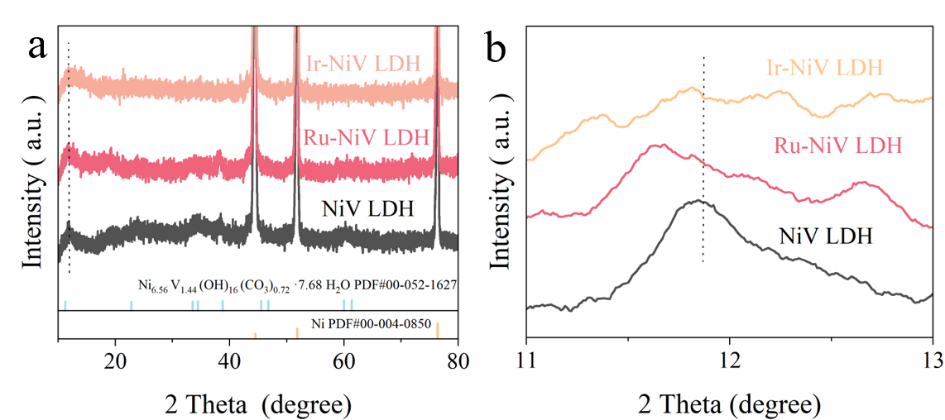


**Fig. S8** **a**) XRD of NiV LDH, Ru-NiV LDH, and Ir-NiV LDH, **b**) the corresponding Fig. S8a enlarges the XRD pattern


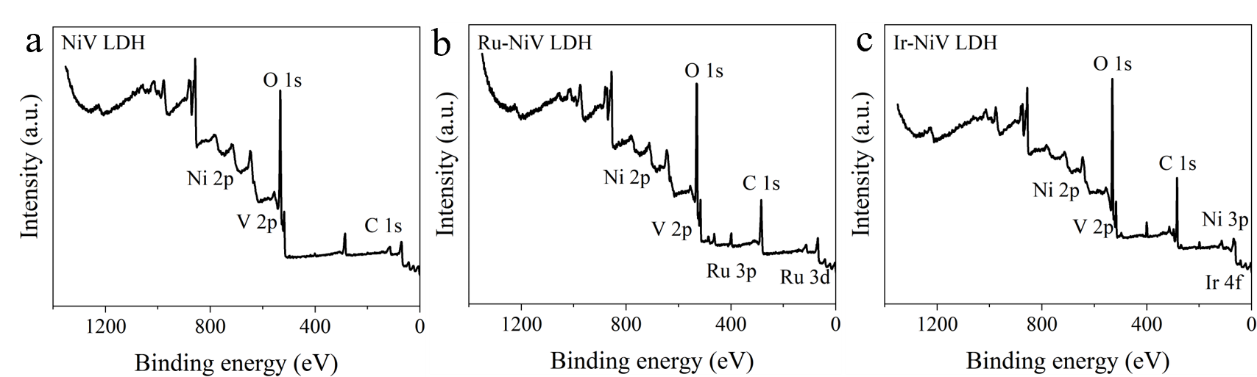


**Fig. S9** XPS survey of **a**) NiV LDH, **b**) Ru-NiV LDH, and **c**) Ir-NiV LDH


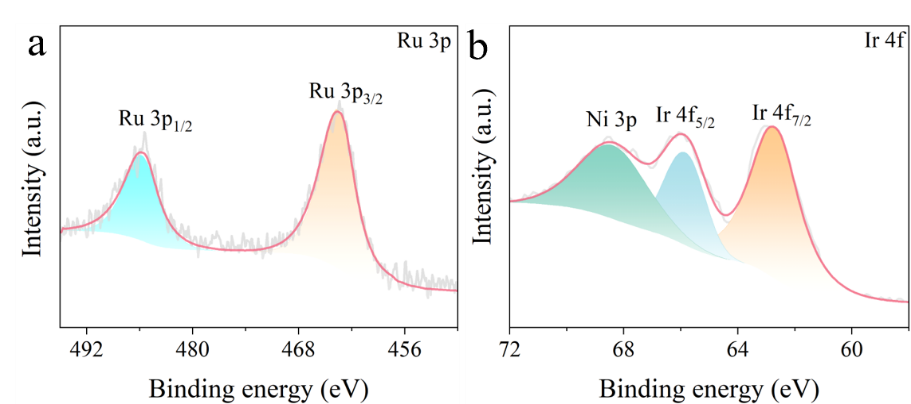


**Fig. S10** **a**) Ru 3p XPS spectra of of Ru-NiV LDH, **b**) Ir 4f XPS spectra of of Ir-NiV LDH


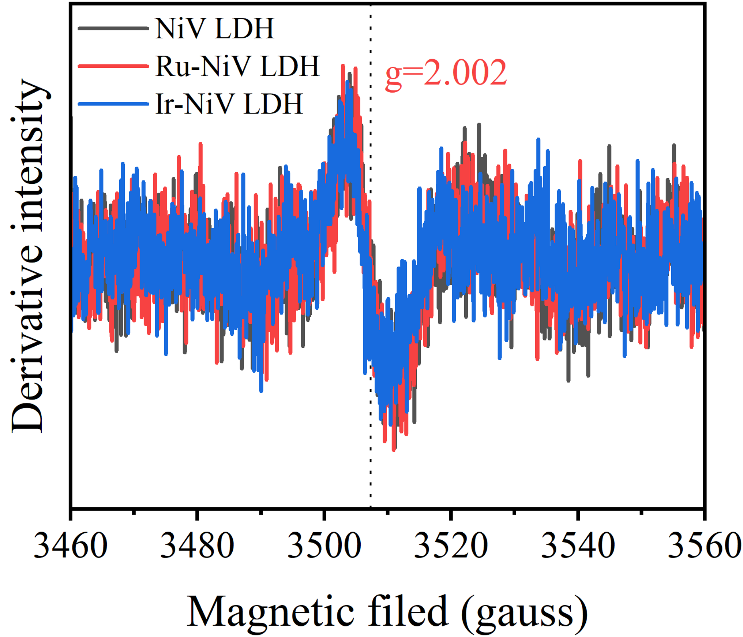


**Fig. S11** EPR of NiV LDH, Ru-NiV LDH, and Ir-NiV LDH(g=2.002)


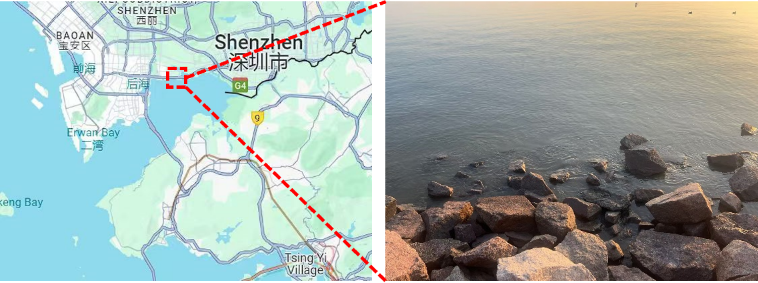


**Fig. S12** The geographical location for the source of natural seawater


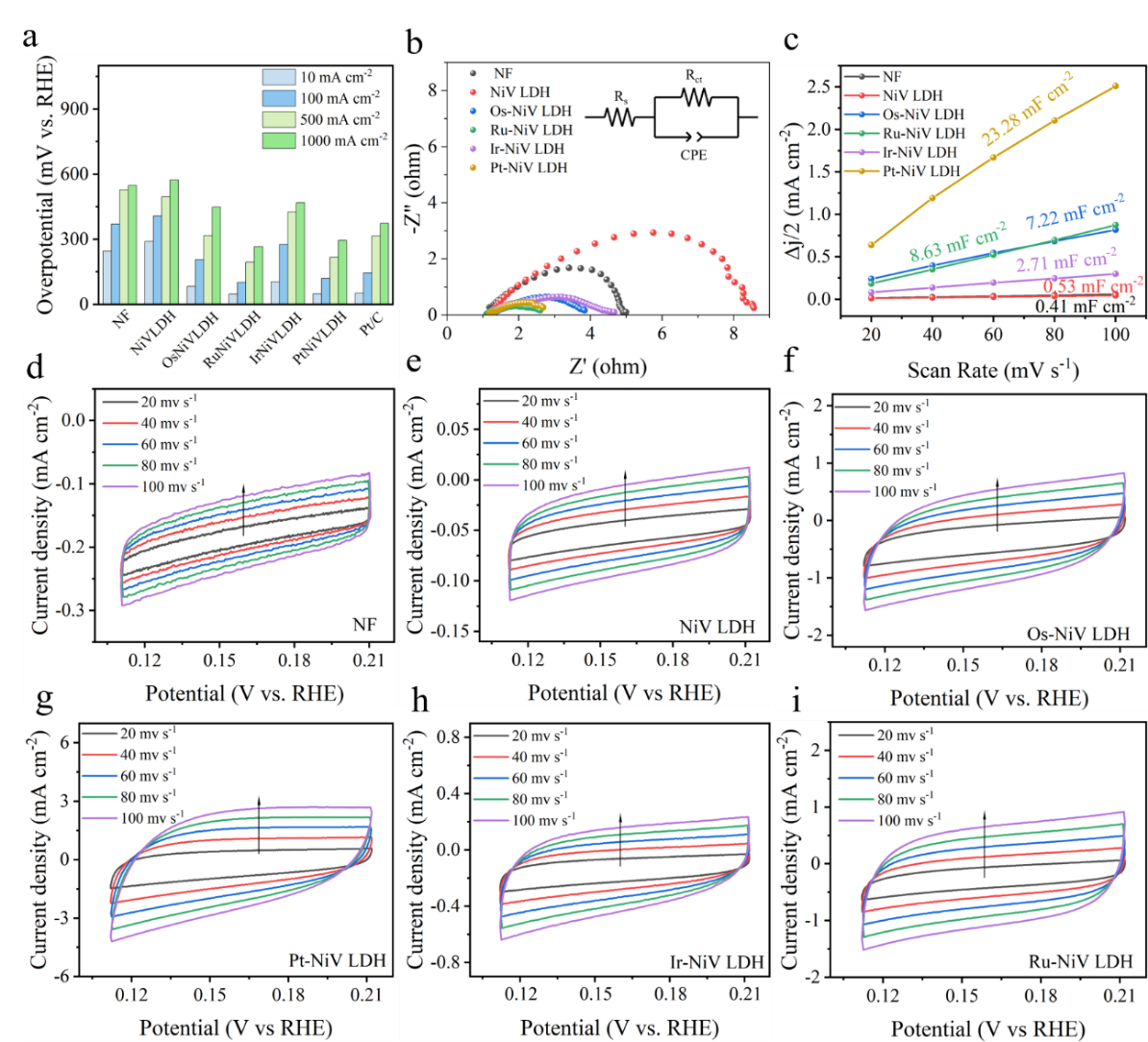


**Fig. S13** HER catalytic performance. **a**) The overpotentials of the catalyst at 10 mA cm^-2^, 100 mA cm^-2^, 500mA cm^-2^, and 1000 mA cm^-2^ were measured, **b**) Nyquist plots (insert of equivalent circuit model), and **c**) the double-layer capacitance of NF, NiV LDH, Os-NiV LDH, Ru-NiV LDH, Ir-NiV LDH, and Pt-NiV LDH, **d-i**) cyclic voltammograms of NF, NiV LDH, Os-NiV LDH, Ru-NiV LDH, Ir-NiV LDH, and Pt-NiV LDH at scan rates in the range of 20-100 mV s^-1^, respectively


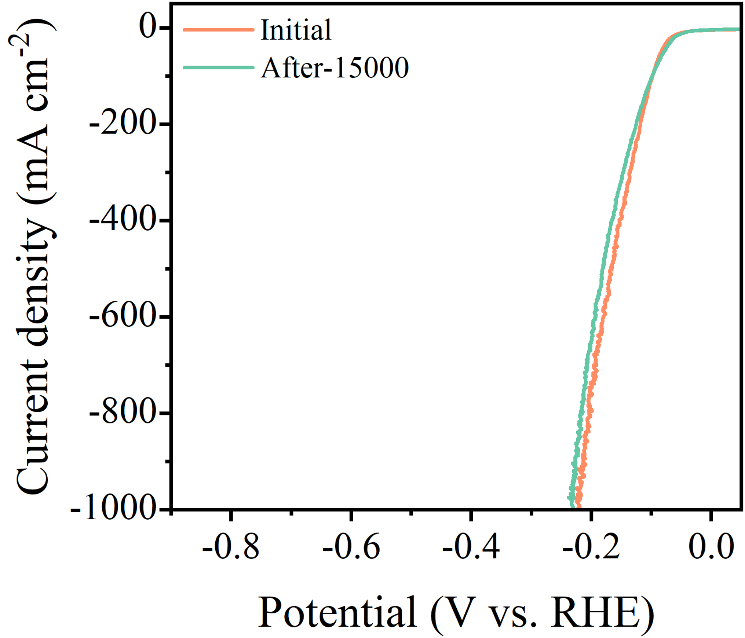


**Fig. S14** Comparison of HER polarization curves before and after 15000 CV cycles


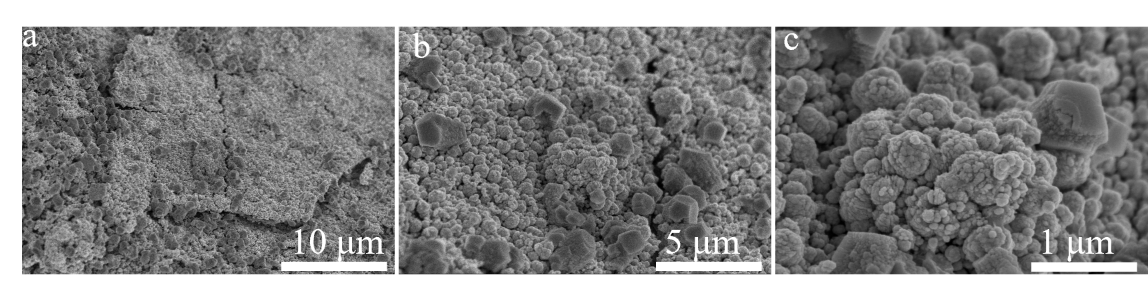


**Fig. S15** SEM images of Ru-NiV LDH after long-term stability for HER


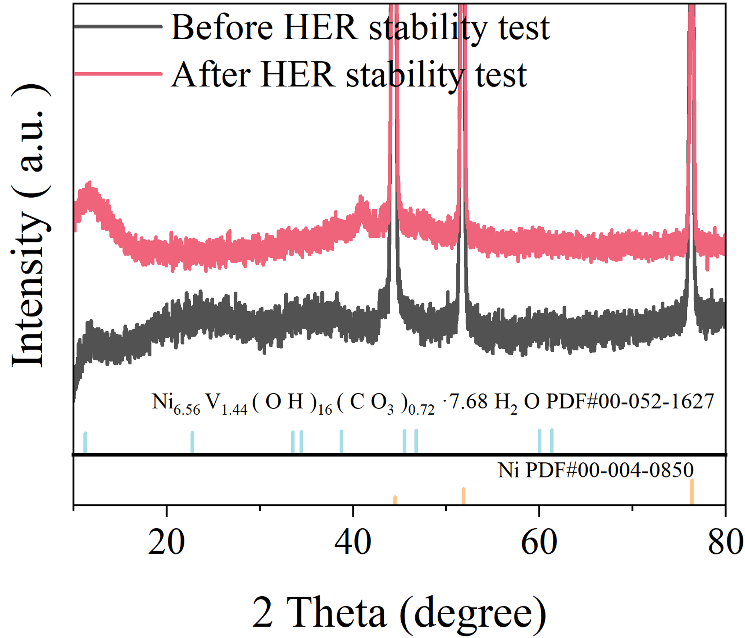


**Fig. S16** XRD pattern of Ru-NiV LDH after long-term stability for HER


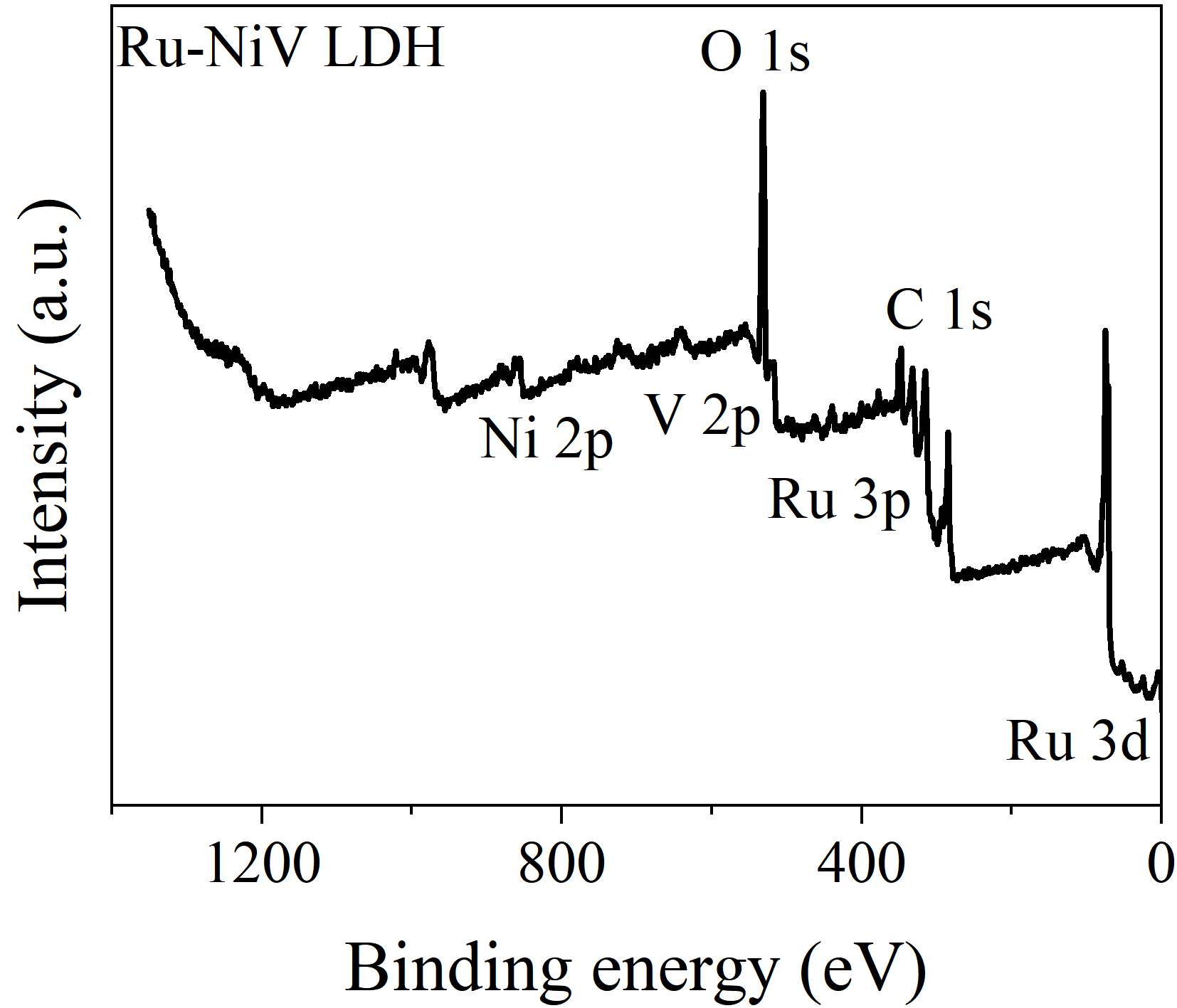


**Fig. S17** XPS survey of Ru-NiV LDH after long-term stability for HER


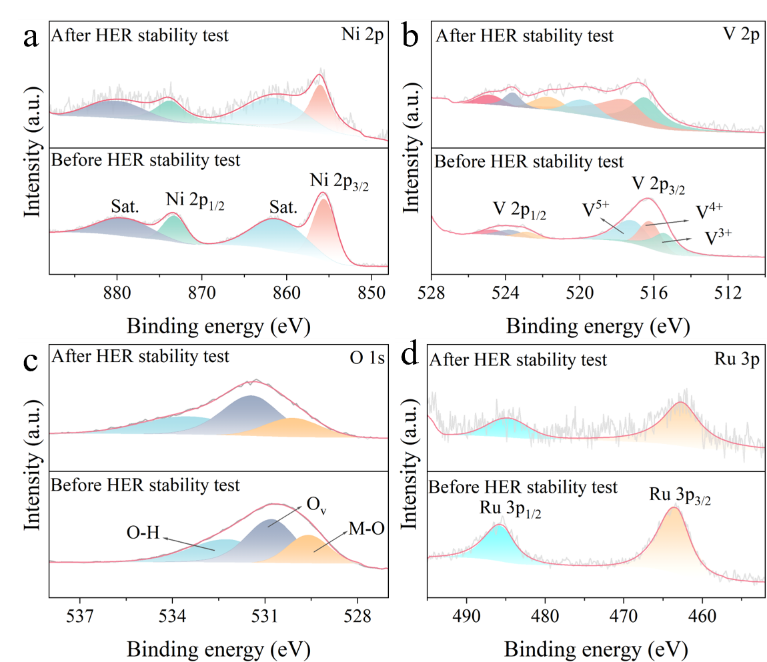


**Fig. S18 a**) Ni 2p, **b**) V 2p, **c**) O 1s, and **d**) Ru 3p of XPS spectra of Ru-NiV LDH after long-term stability for HER


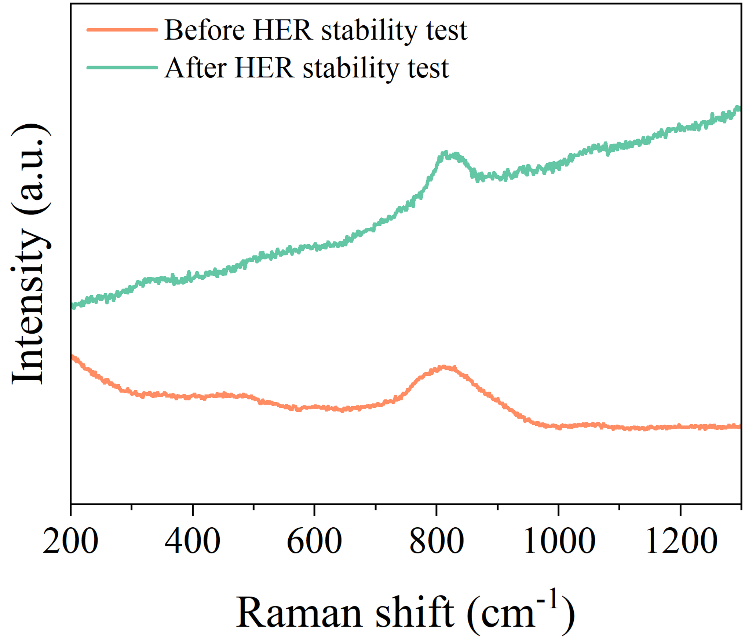


**Fig. S19** Comparison of Raman spectra results for Ru-NiV LDH before and after long-term HER stability testing


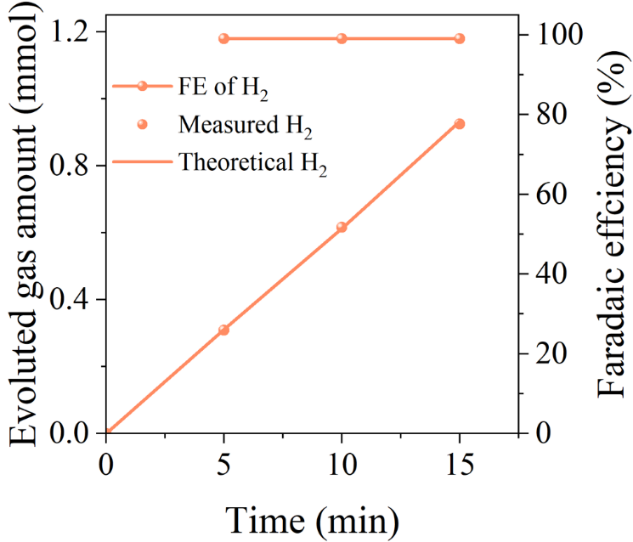


**Fig. S20** Experimental and theoretical gaseous products (H_2_ and O_2_) by the two-electrode electrolyzer at a constant current density of 200 mA cm^-2^, optical images of drainage method. Experimental and theoretical gaseous products (H_2_) by the two-electrode electrolyzer


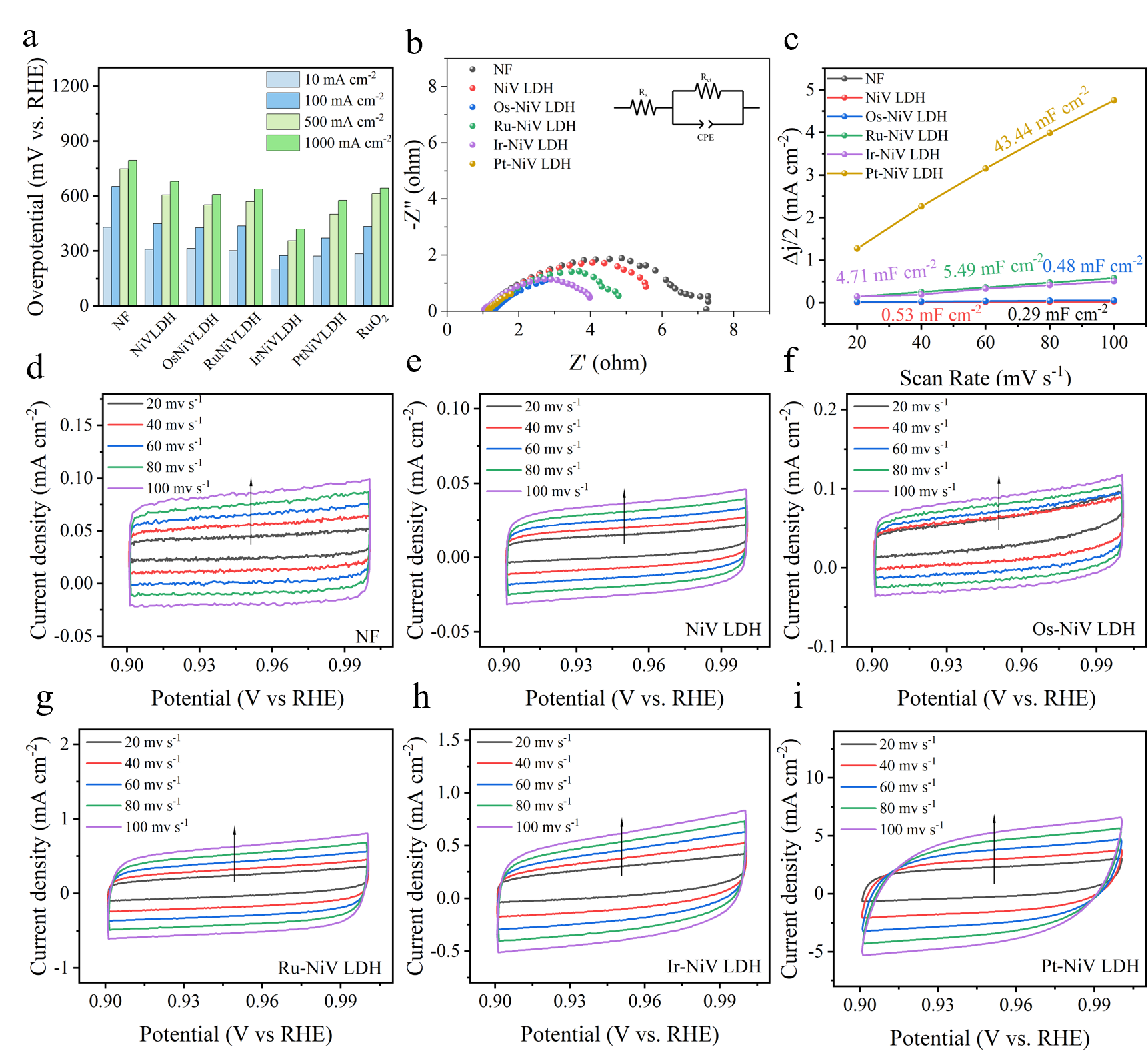


**Fig. S21** OER catalytic performance. **a**) The overpotentials of the catalyst at 10 mA cm^-2^, 100 mA cm^-2^, 500mA cm^-2^, and 1000 mA cm^-2^ were measured, **b**) Nyquist plots (insert of equivalent circuit model), and **c**) the double-layer capacitance of NF, NiV LDH, Os-NiV LDH, Ru-NiV LDH, Ir-NiV LDH, and Pt-NiV LDH, **d-i**) cyclic voltammograms of NF, NiV LDH, Os-NiV LDH, Ru-NiV LDH, Ir-NiV LDH, and Pt-NiV LDH at scan rates in the range of 20-100 mV s^-1^, respectively


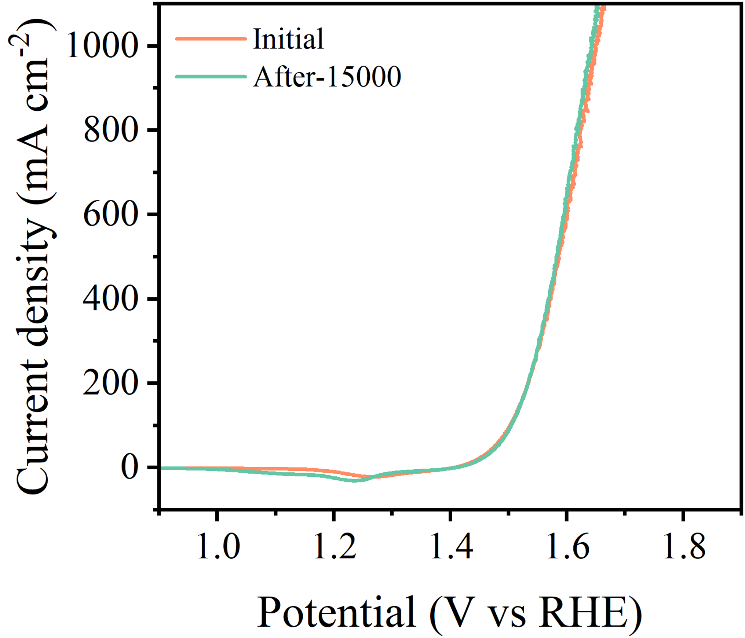


**Fig. S22** Comparison of OER polarization curves before and after 15000 CV cycles


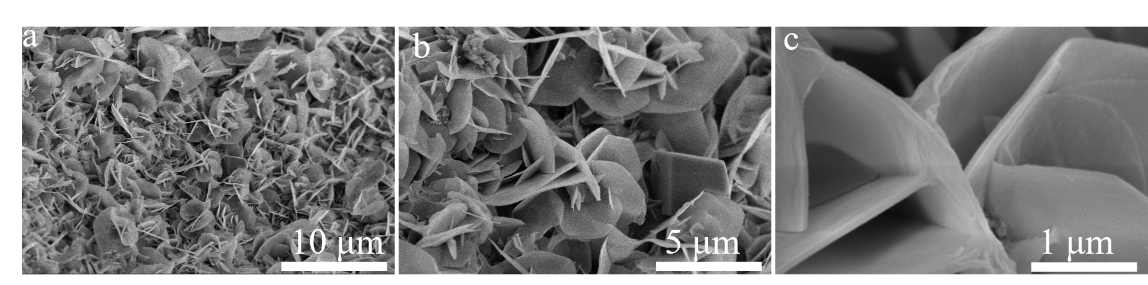


**Fig. S23** SEM images of Ir-NiV LDH after long-term stability for OER


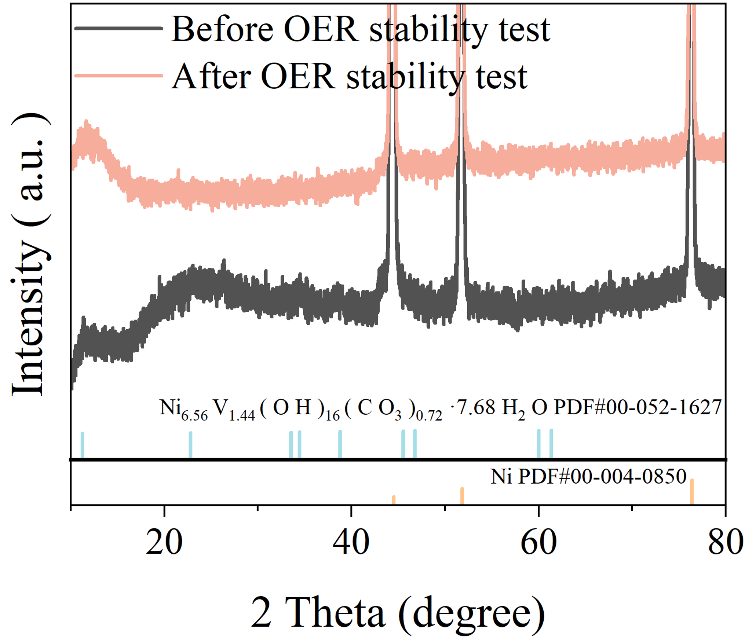


**Fig. S24** XRD pattern of Ir-NiV LDH after long-term stability for OER


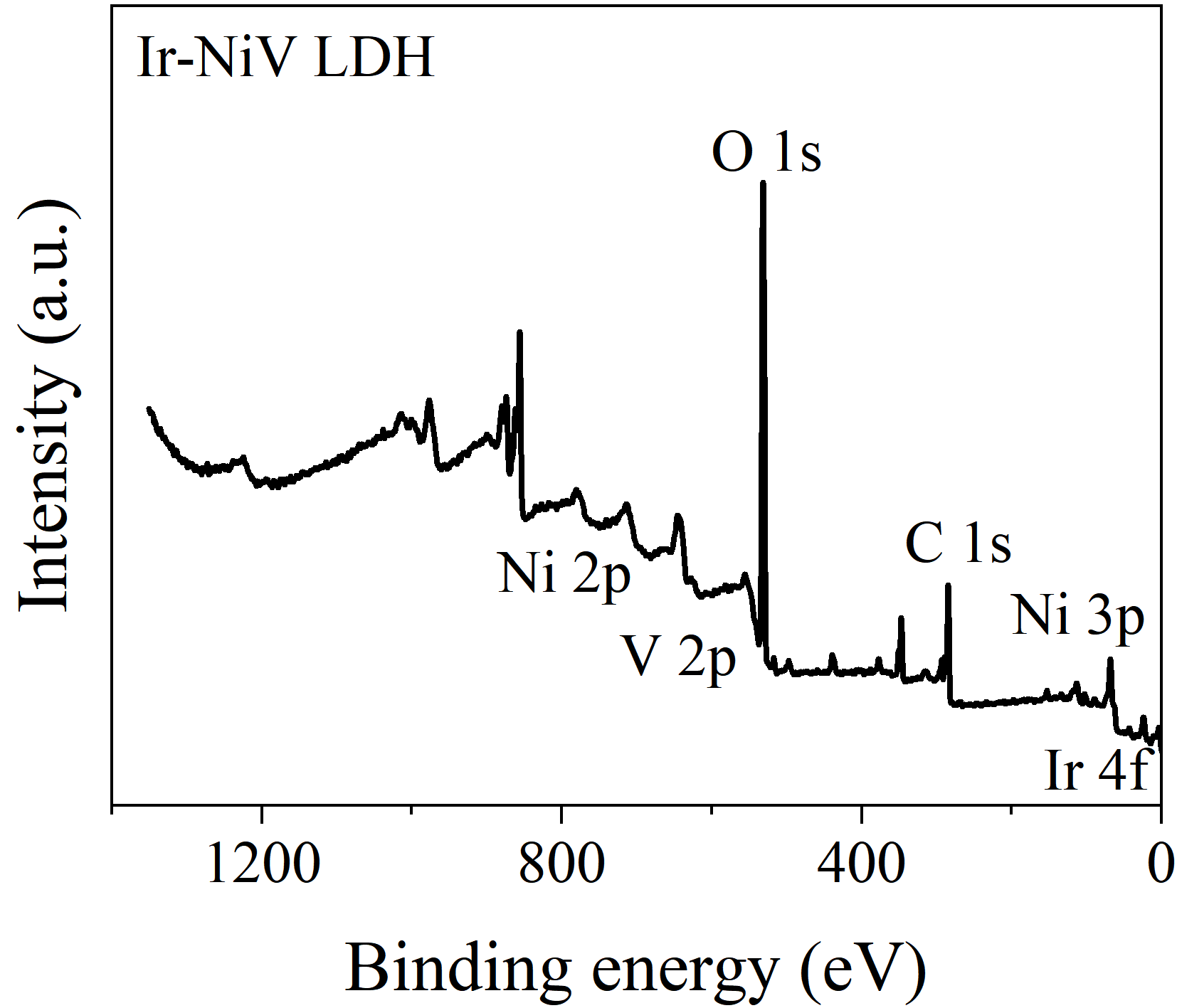


**Fig. S25** XPS survey of Ir-NiV LDH after long-term stability for OER


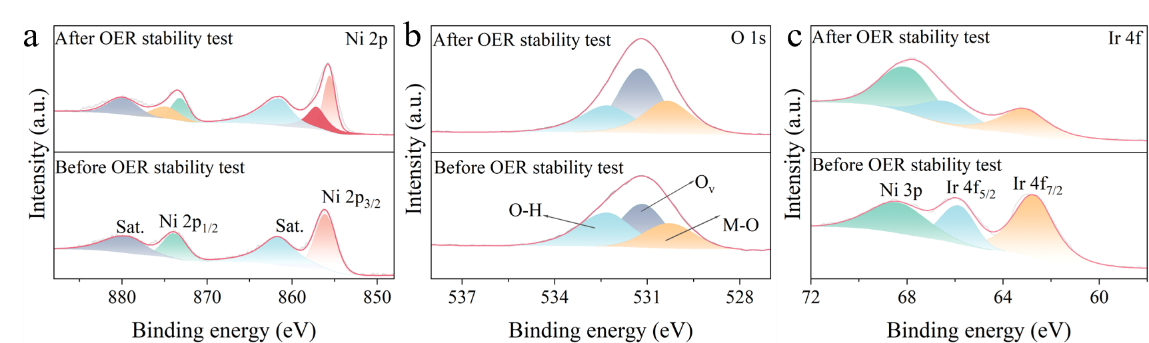


**Fig. S26** **a**) Ni 2p, **b**) O 1s, and **c**) Ir 4f of XPS spectra of Ir-NiV LDH after long-term stability for OER


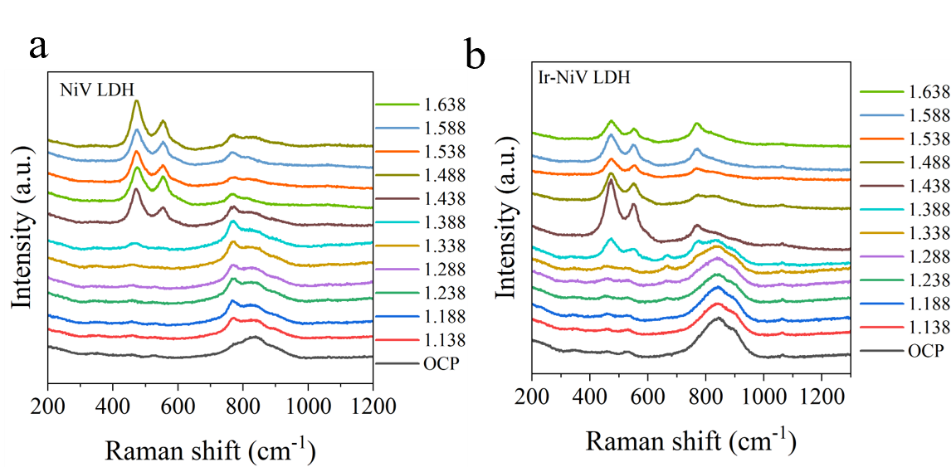


**Fig. S27** **a-b**) In-situ Raman **spectrum** of NiVLDH and Ir-NiVLDH in 1 M KOH, respectively


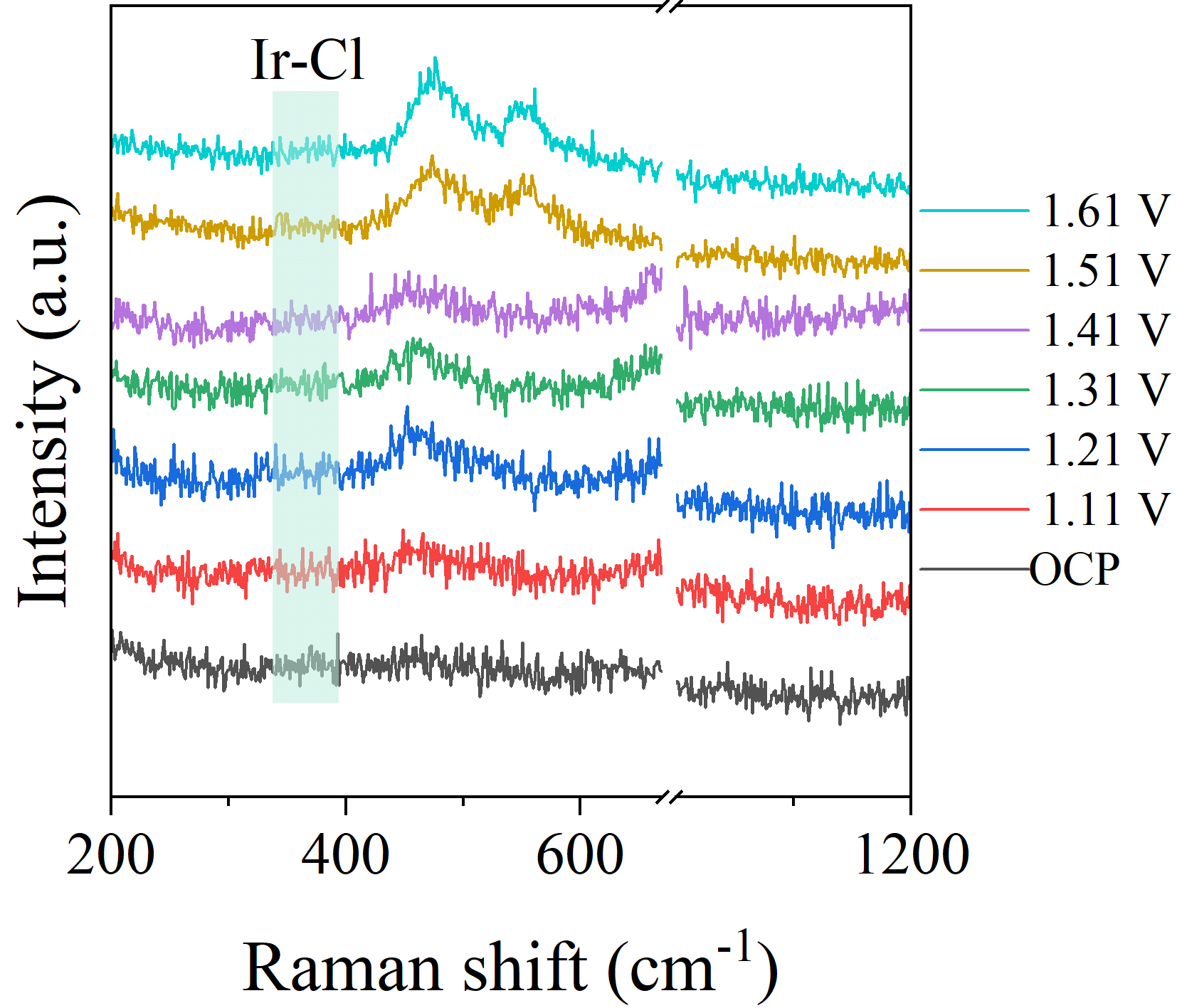


**Fig. S28** In-situ Raman **spectrum** of Ir-NiVLDH in 1 M KOH + 0.5 M NaCl


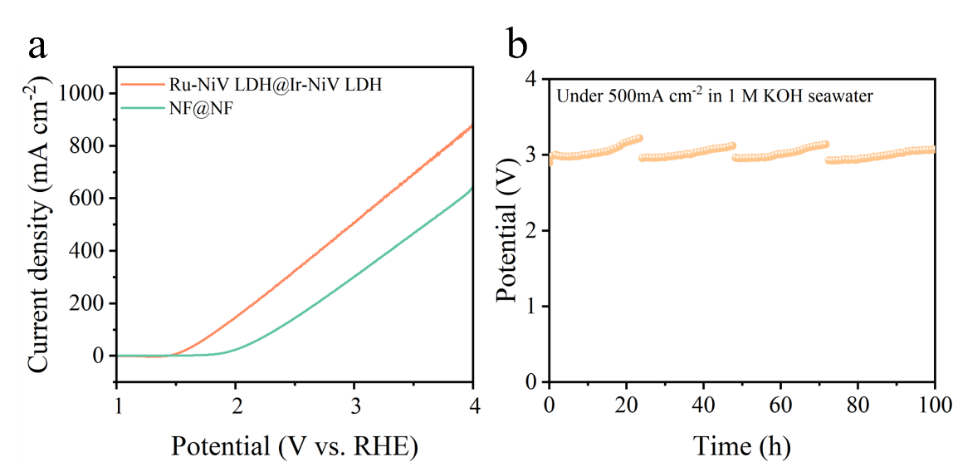


**Fig. S29** The total water splitting performance (without iR compensation) of the two-electrode system in alkaline seawater electrolyte. **a**) Polarization curve, and **b**) durability test at an industrial-grade current density of 500 mA cm^-2^


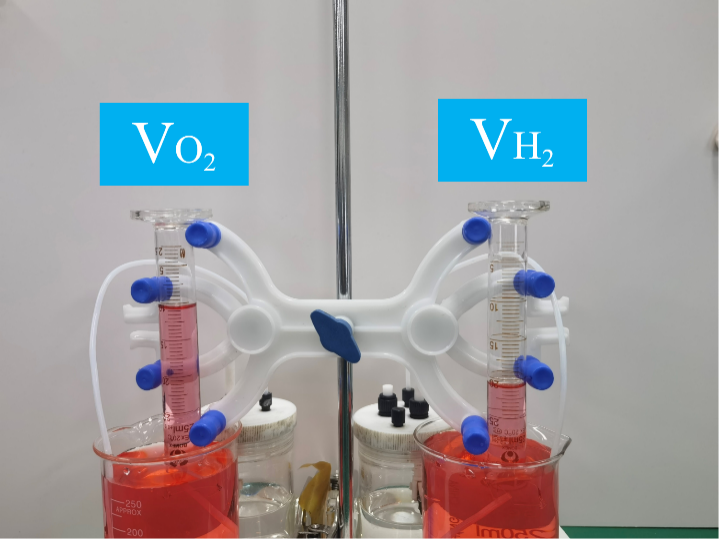


**Fig. S30** Experimental and theoretical gaseous products (H_2_ and O_2_) by the two-electrode electrolyzer at a constant current density of 200 mA cm^-2^, optical images of drainage method. Photographs of collected hydrogen and oxygen


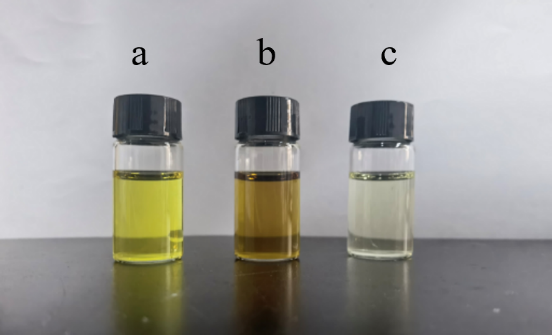


**Fig. S31** O-tolidine test for NiV LDH (**a**), Ru-NiV LDH (**b**), and Ir-NiV LDH (**c**). Photographs of the testing solutions


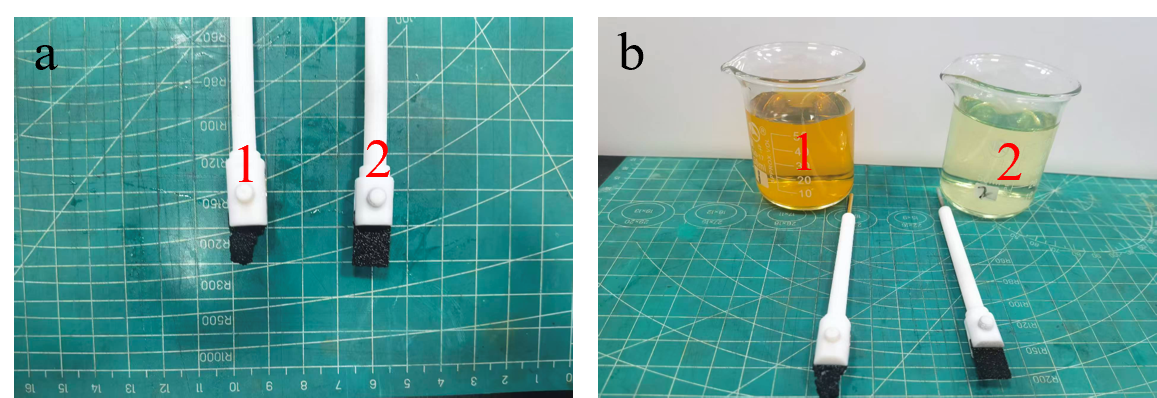


**Fig. S32** **a**) Photographs of the NiV LDH (1) and Ir-NiV LDH (2) catalysts after the chronoamperometry (i-t) test in a 1 M KOH + 0.5 M NaCl electrolyte; **b**) Corresponding images of ClO⁻ detection in an o-phenylenediamine solution


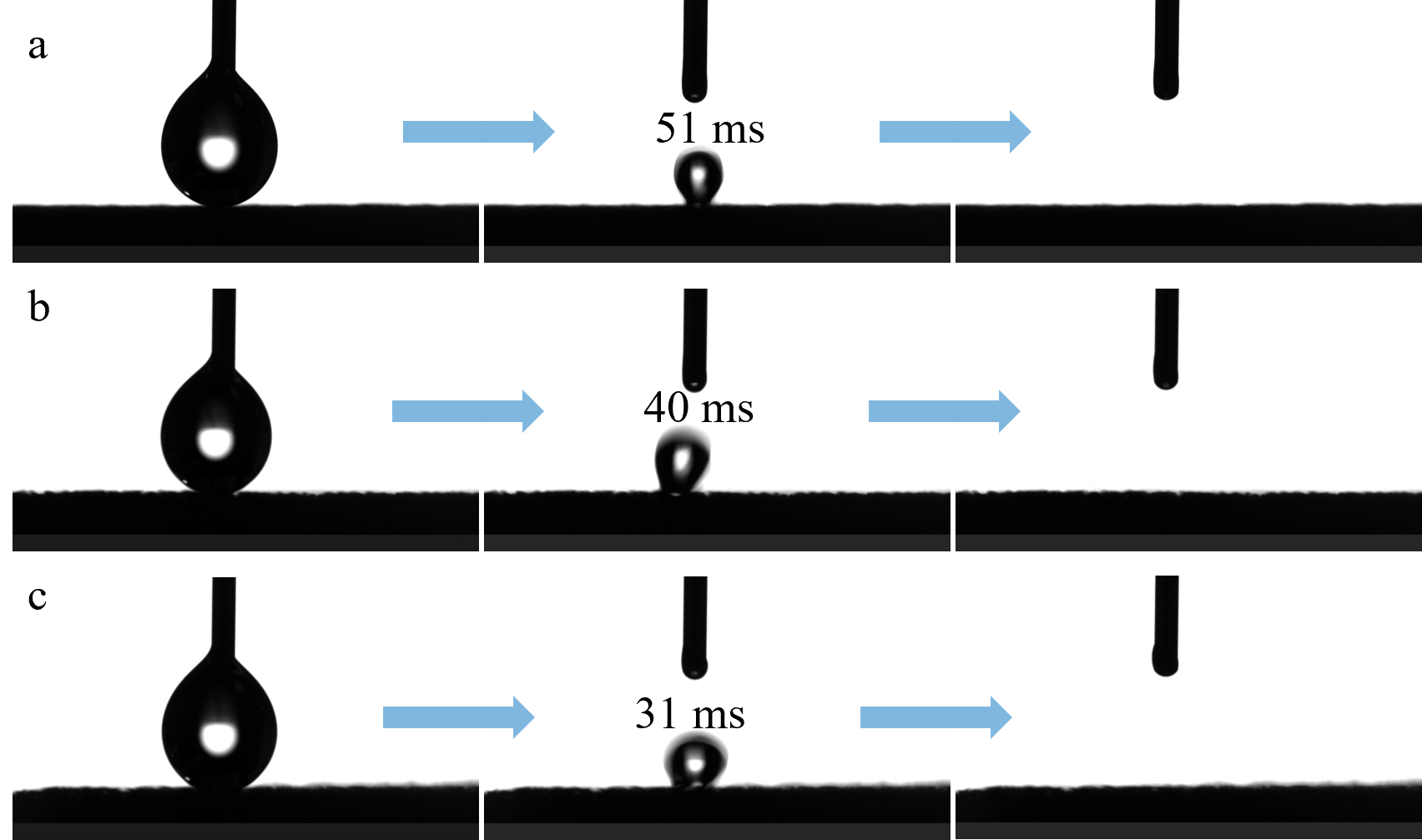


**Fig. S33** Water contact angles of **a**) NiV LDH, **b**) Ru-NiV LDH, and **c**) Ir-NiV LDH


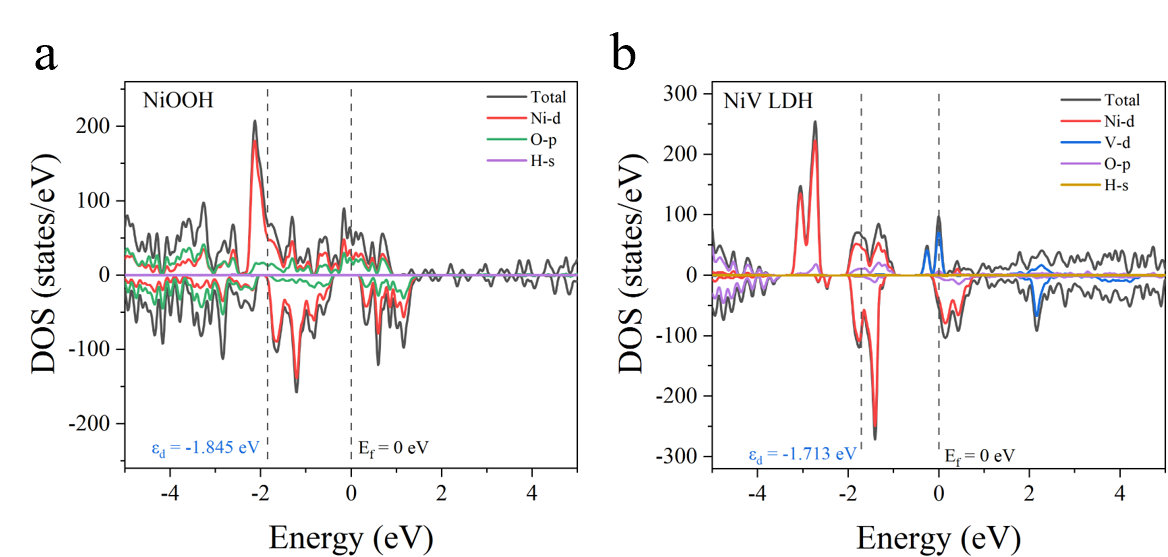


**Fig. S34** **a**) The projected density of states of NiOOH, **b**) the projected density of states of NiV LDH


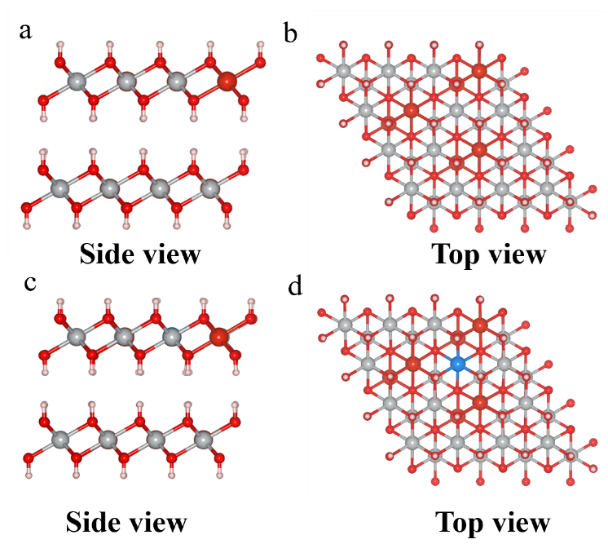


**Fig. S35** Theoretical models of NiV LDH and Ru-NiV LDH. **a**) Side view of NiV LDH models, **b**) top view of NiV LDH models, **c**) side view of Ru-NiV LDH models, d) top view of Ru-NiV LDH models


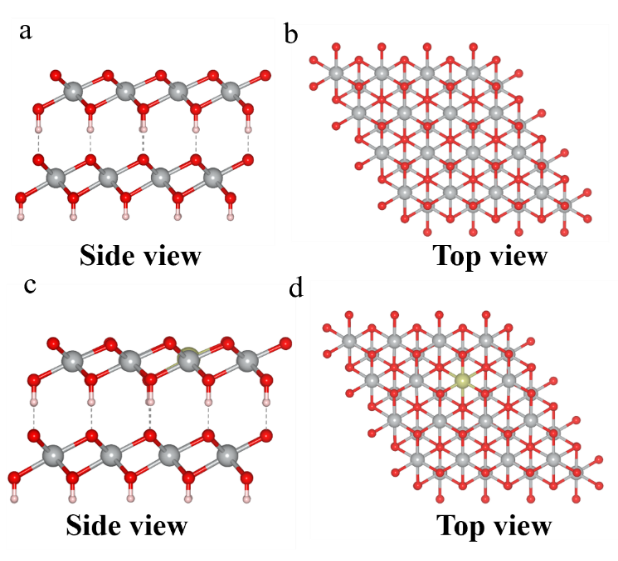


**Fig. S36** Theoretical models of NiOOH and Ir-NiOOH. **a**) Side view of NiOOH models, **b**) top view of NiOOH models, **c**) side view of Ir-NiOOH models, **d**) top view of Ir-NiOOH models


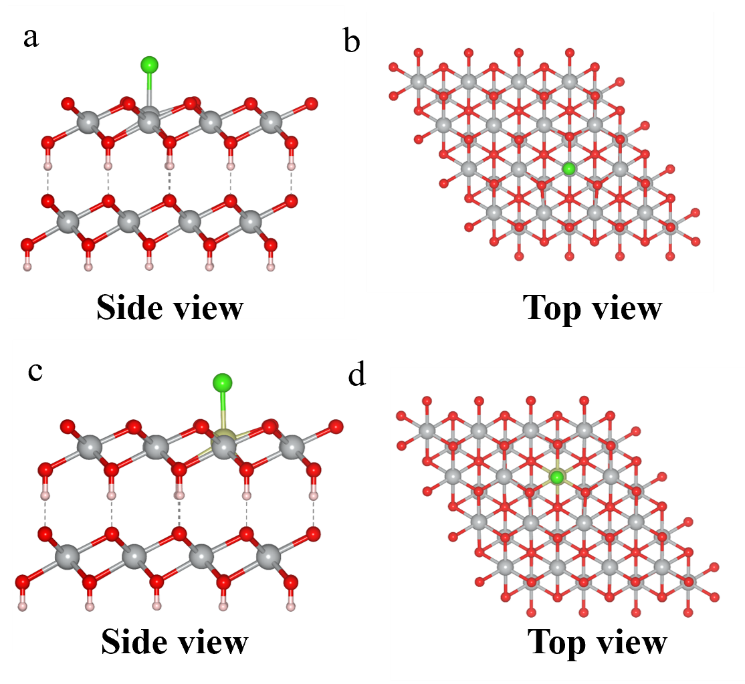


**Fig. S37** The theoretical model of Cl^-^ adsorption on NiOOH and Ir-NiOOH. **a**) The side view of Cl^-^ adsorbed by NiOOH model, **b**) top view of Cl^-^ adsorption on NiOOH model, **c**) Ir-NiOOH model adsorption Cl^-^ side view, **d**) Ir-NiOOH mode adsorption Cl^-^ type top view


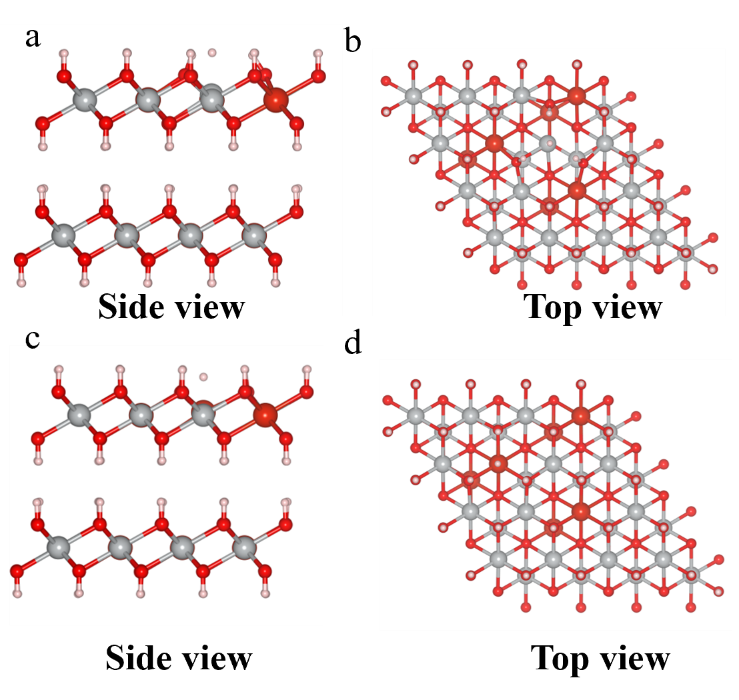


**Fig. S38 a**) Ni site side view, **b**) Ni site top view, **c**) V site side view, **d**) V site top view of NiV LDH and the possible adsorption H * configuration


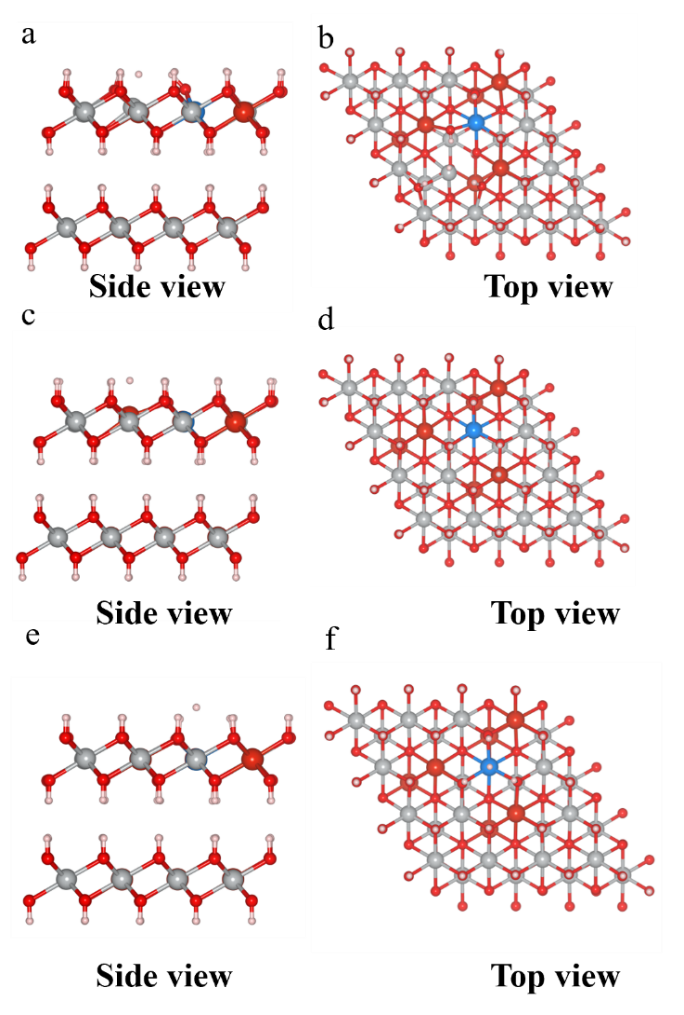


**Fig. S39 a**) Ni site side view, **b**) Ni site top view, **c**) V site side view, **d**) V site top view, **e**) Ru site side view, **f**) Ru site top view of Ru-NiV LDH and the possible adsorption H * configuration


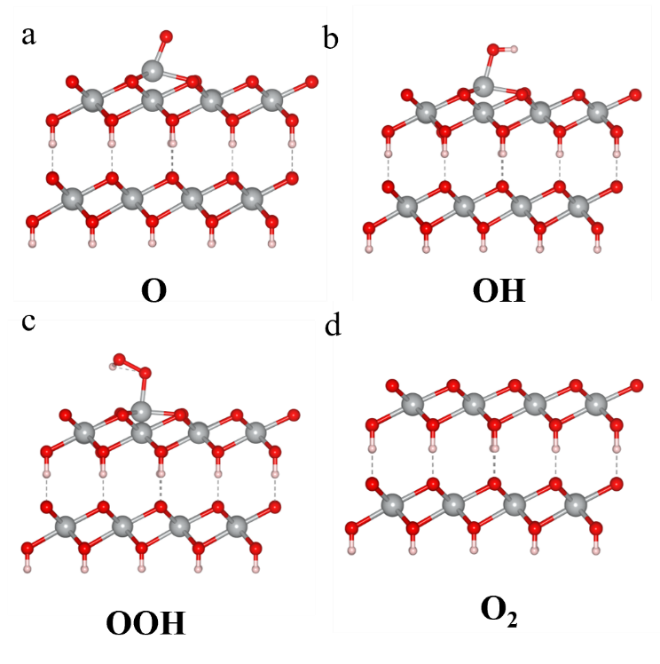


**Fig. S34** Reaction pathway for OER on NiOOH of Ni site. **a**) O* process, **b**) OH* process, **c**) OOH* process, **d**) O_2_ process


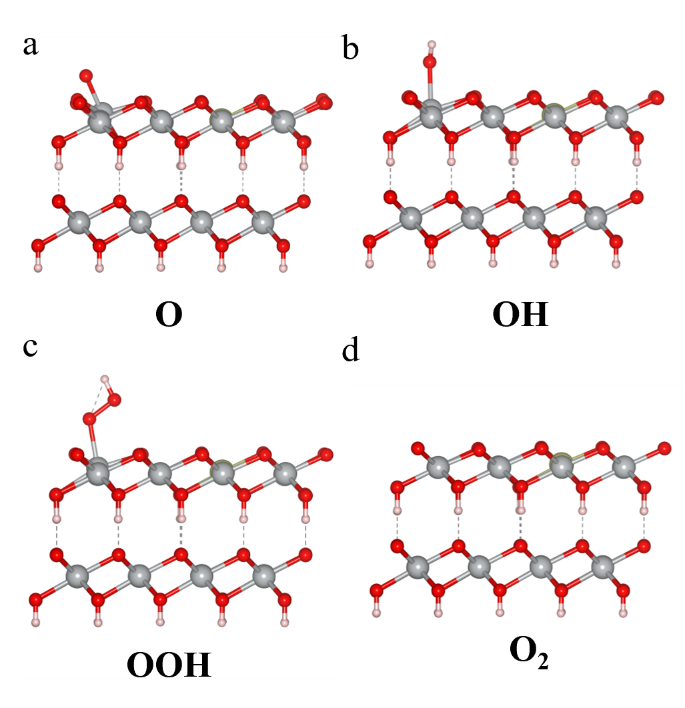


**Fig. S41** Reaction pathway for OER on Ir-NiOOH of Ni site. **a**) O* process, **b**) OH* process, **c**) OOH* process, **d**) O_2_ process


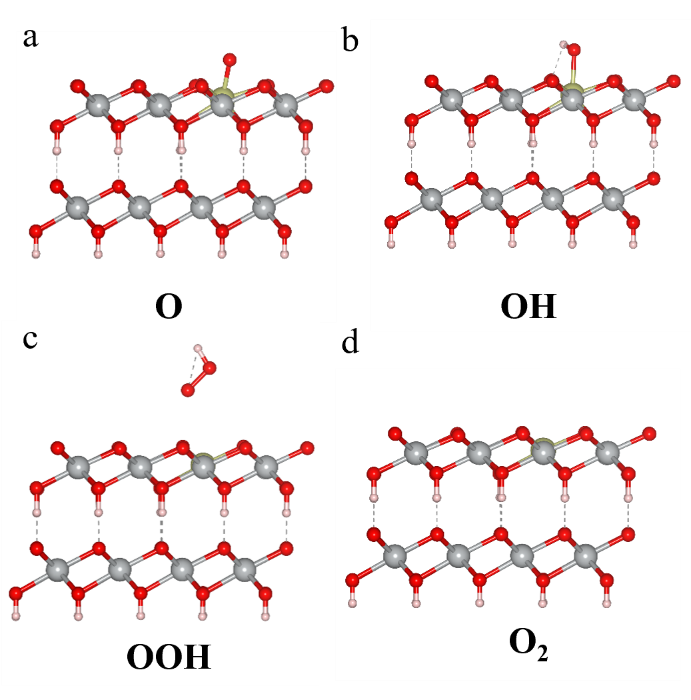


**Fig. S42** Reaction pathway for OER on Ir-NiOOH of Ir site. **a**) O* process, **b**) OH* process, **c**) OOH* process, **d**) O_2_ process


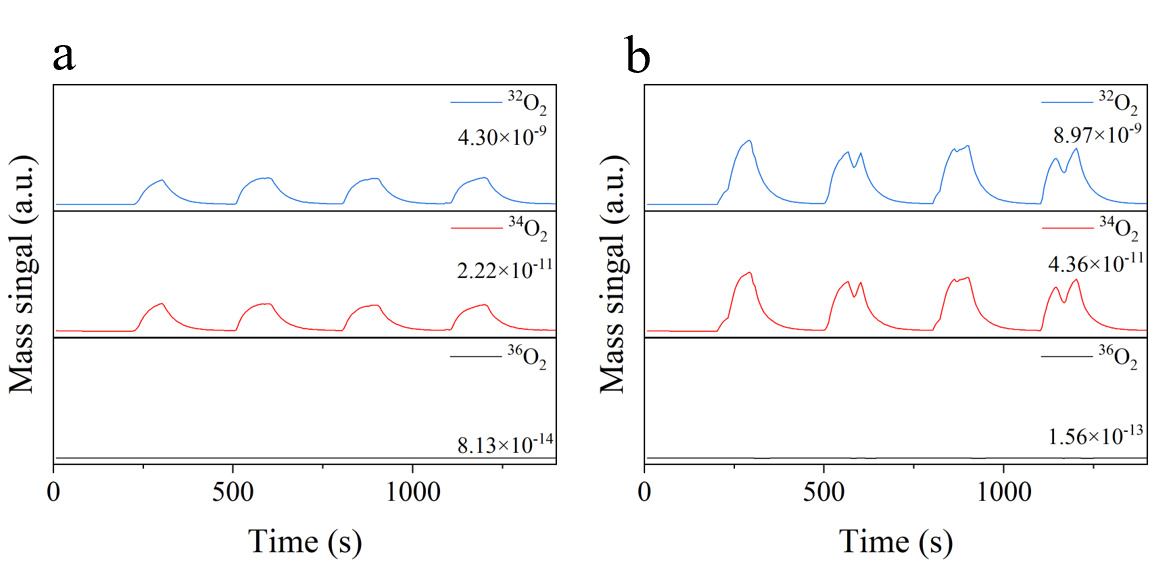


**Fig. S43** Oxygen production results. **a**) The results of DEMS for 18O-labeled NiV LDH, **b**) The results of DEMS for 18O-labeled Ir-NiV LDH

**Table S1** ICP-OES results of pristine NiV LDH, Ru-NiV LDH and Ir-NiV LDH

|  | Element | Mass Fraction |
| --- | --- | --- |
| NiV LDH | Ni | 85.69% |
|  | V | 3.62 % |
| Ru-NiV LDH | Ni | 93.93 % |
|  | V | 1.73 % |
|  | Ru | 0.23 % |
| Ir-NiV LDH | Ni | 88.03% |
|  | V | 1.75 % |
|  | Ir | 0.68 % |

**Table S2** ICP-OES for untested liquids, HER stability test liquids and OER stability test results

|  | Element | Element content  （mg/L) |
| --- | --- | --- |
| 1M KOH+Seawater | K | 29795.40 |
|  | Na | 1093.19 |
|  | V | <0.2 |
| HER Stability Test  1M KOH+Seawater | K | 54190.40 |
|  | Na | 4025.87 |
|  | V | 1.69 |
| OER Stability Test  1M KOH+Seawater | K | 53953.40 |
|  | Na | 4109.17 |
|  | V | 1.67 |

**Table S3** The stability current density and stability time of Ru-NiV LDH and the most advanced HER catalyst in alkaline seawater were compared

| Electrocatalysts | Current density (mA cm^-2^) | Time (h) | References |
| --- | --- | --- | --- |
| Ru-NiV LDH | 500 | 2350 | This work |
| Fe_3_Se_4_/NiSe_2_@MXene | 500 | 140 | Adv. Funct. Mater. 2025, 35, 2424718 |
| caMo-NiFePO/NMF | 500 | 480 | Adv. Funct. Mater. 2025, 35, 2504862 |
| Ru-Ni_2_P/Fe_2_P | 500 | 100 | Adv. Funct. Mater. 2024, 34, 2400734 |
| Pt@Ni_2_P_v_/NF | 1000 | 1200 | Adv. Funct. Mater. 2025, e14592 |
| NiP_2_@CoP | 400 | 400 | Nano Research, 2025, 18, 9490706 |
| Fe-Ni_2_Pv | 100 | 100 | Adv. Mater. 2024, 36, 2307395 |
| Ru@NMoC | 500 | 500 | Angew. Chem. Int. Ed. 2025, 64, e202505031 |
| HW-NiMoN | 500 | 100 | Nano-Micro Lett. 15, 2023, 157 |
| Ni-MoN | 500 | 100 | Adv. Mater. 2022, 34, 2201774 |
| MnCo/NiSe | 500 | 200 | Appl. Catal. B Environ. 2023, 325, 122355 |
| Fe_4_N/Co_3_N/MoO_2_ | 500 | 200 | Adv. Mater. 2024, 36, 2405852 |
| RuMo/Cu_2_O@C | 500 | 300 | Adv. Mater. 2025, 37, 2416658 |
| Am-NiMoB | 500 | 350 | Appl. Catal. B Environ. 2025, 365, 124928 |
| D-Ni_3_Mo/NF | 500 | 100 | Chem. Eng. J.2024, 485, 150044 |
| Pt-SAs/ac-NiFe LDH | 500 | 100 | J. Energy Chem. 2025,107, 427–439 |

**Table S4** The stability current density and stability time of Ir-NiV LDH and the most advanced OER catalyst in alkaline seawater were compared

| Electrocatalysts | Current density (mA cm^-2^) | Time (h) | References |
| --- | --- | --- | --- |
| Ir-NiV LDH | 500 | 2750 | This work |
| Fe_3_Se_4_/NiSe_2_@MXene | 500 | 130 | Adv. Funct. Mater. 2025, 35, 2424718 |
| caMo-NiFePO/NMF | 500 | 480 | Adv. Funct. Mater. 2025, 35, 2504862 |
| Ru-Ni_2_P/Fe_2_P | 500 | 100 | Adv. Funct. Mater. 2024, 34, 2400734 |
| CeNiFe@CuO | 500 | 300 | Adv. Funct. Mater. 2025, 35, 2508539 |
| BZ-NiFe-LDH/CC | 500 | 100 | Nano Research Energy 2022, 1, e9120028 |
| Cr-NiFeLDH | 100 | 100 | Appl. Catal. B Environ. 2026, 383, 126109 |
| NiP_2_@CoP | 400 | 400 | Nano Research, 2025, 18, 9490706 |
| Fe-Ni_2_Pv | 100 | 100 | Adv. Mater. 2024, 36, 2307395 |
| Ni_0.10_-Fe_3_N@NCPs | 500 | 200 | Adv. Funct. Mater. 2024, 34, 2404470 |
| MnCo/NiSe | 500 | 200 | Appl. Catal. B Environ. 2023, 325, 122355 |
| Fe_4_N/Co_3_N/MoO_2_ | 500 | 200 | Adv. Mater. 2024, 36, 2405852 |
| HEA-Mo_2_C | 500 | 1000 | Adv. Funct. Mater. 2025, e17862 |
| MCF-LDH | 500 | 600 | Adv. Energy Mater. 2025, 15, e03465 |
| NiFe LDH_CO_3_^2-^ | 500 | 1000 | Adv. Energy Mater. 2024, 14, 2400053 |
| Ce(OH)_3_@NiFe LDH | 1000 | 1000 | Small 2025, 21, 2505219 |
